# Epidemiological and sociodemographic transitions in the global burden and risk factors for Alzheimer's disease and other dementias: a secondary analysis of GBD 2021

**DOI:** 10.1186/s12939-025-02530-2

**Published:** 2025-05-24

**Authors:** Changqing Xu, Chuanping Jiang, Xiaoxue Liu, Wenqi Shi, Jianjun Bai, Sumaira Mubarik, Fang Wang

**Affiliations:** 1 Department of Biostatistics, School of Public Health, Xuzhou Medical University, Xuzhou, 221004 Jiangsu China; 2 Department of Outpatient Office, The First People’s Hospital of Xuzhou, The Affiliated Xuzhou Municipal Hospital of Xuzhou Medical University, Xuzhou, 221100 Jiangsu China; 3 Global Health Research Division, Public Health Research Center and Department of Public Health and Preventive Medicine, Wuxi School of Medicine, Jiangnan University, Wuxi, 214122 Jiangsu China; 4 School of Public Health, Peking University, Beijing, 100871 China; 5 PharmacoTherapy,-Epidemiology and-Economics, Groningen Research Institute of Pharmacy, University of Groningen, Groningen, The Netherlands; 6 Key Laboratory of Human Genetics and Environmental Medicine, Xuzhou Medical University, Xuzhou, 221004 Jiangsu China; 7 Jiangsu Engineering Research Center of Biological Data Mining and Healthcare Transformation, Xuzhou Medical University, Xuzhou, 221004 Jiangsu China

**Keywords:** Global burden of disease, Alzheimer's disease and other dementias, Socio-demographic index, Risk factors, Trends

## Abstract

**Background:**

The study aimed to analyze the long-term trends in the global burden of Alzheimer's disease and other dementias(ADOD) in different regions, and assess the association between socio-demographic index(SDI) and disease burden.

**Methods:**

We extracted data on the incidence, mortality, disability-adjusted life-years(DALYs), and age-standardized rates related to ADOD, as disease burden measures from 1990 to 2021. The joinpoint regression, quantile regression and restricted cubic splines were adopted to estimate the temporal trends and relationships with SDI. Risk factors for deaths and DALYs were also analyzed.

**Results:**

Globally, 9.84 million cases of ADOD occurred in 2021, with 1.95 million ADOD-related deaths, causing 36.33 million DALYs. ADOD incidence, mortality and DALYs all increased from 1990 to 2021. Regional and sex variations persisted, with the fastest increase in age-standardized death rate in low-middle SDI quintiles, experienced the highest estimated annual percentage changes (0.41[0.31,0.52]). The incidence of ADOD increased more rapidly as SDI increased in areas that have historically shown lower incidence compared to other areas. In regions with higher mortality or DALYs burden, these indicators decreased relatively faster as SDI increased. High fasting plasma glucose was the main risk factor, particularly in high SDI region, with an increasing trend in attributable burden. The burden attributable to high BMI was increasing, whereas the burden associated with smoking steadily decreased.

**Conclusion:**

ADOD poses a significant and escalating challenge to healthcare sustainability, with persistent regional and gender disparities. By learning from successful ADOD management in certain nations, we can proactively reduce health burdens and bridge disparities between countries at various developmental levels.

**Supplementary Information:**

The online version contains supplementary material available at 10.1186/s12939-025-02530-2.

## Introduction

As life expectancy increases, age-related neurological disorders such as stroke, Alzheimer's disease and other dementias (ADOD) are also on the rise. ADOD can affect individuals'memory, thinking, behavior, and emotions, subsequently hindering their ability to perform daily activities [1]. As a result, people living with ADOD often experience a loss of independence and the ability to perform activities of daily living. The economic burden associated with ADOD is quite heavy, with a global economic loss of $1.3 trillion in dementia in 2019, of which approximately 50% of the cost is attributed to the informal caregivers such as family members and close friends, who may reduce their working hours while caring for people with dementia, leading to a loss of productivity [2, 3].

According to the World Health Organization, there were more than 50 million people living with dementia worldwide, and more than 60% of whom live in low- and middle-income countries [2]. As societies age, this number is expected to increase to 139 million by 2050 according to World Alzheimer Report [4]. 2023 Alzheimer's disease facts and figures indicated that the number of people with Alzheimer's disease aged 65 and older might increase greatly from 6.7 million to 13.8 million by 2050 in America [5]. Data suggested that the prevalence of dementia will double in Europe and triple globally by 2050 [6]. The prevalence of cardiovascular and cerebrovascular disease also increases the risk of ADOD. The increase in the number of people with ADOD means that substantial financial resources are needed to effectively provide medical care, thus increasing the economic burden. Additionally, the ongoing care and treatment related to ADOD can place significant financial strain on individuals, their support networks, and society [7].

As the most common form of dementia, there are no effective treatments for ADOD, but adopting healthy lifestyle behaviours [8, 9], such as regular physical activity, and increased social and cognitive engagement [5, 10, 11], may reduce the risk of developing cognitive decline or ADOD. The 2024 report of the Lancet Commission suggested that nearly half of all dementia cases could theoretically be prevented by eliminating 14 possible risk factors [12]. One quarter of countries worldwide have implemented policies and programs regarding dementia, with approximately half of them situated in the European region [2]. Although some regions have made progress, disparities in disease burden still persist across regions, particularly among gender groups [7, 13]. In underdeveloped countries, demographic transitions and increasing urbanization can lead to changes in local lifestyles, which increases the risk of ADOD [14]. ADOD disproportionately affects women, who are more likely to develop ADOD, and women are also tending to be the primary caregivers of families, thus potentially placing a double burden on this population [13]. The disease burden, risk factors, and preventive measures of ADOD may vary in different regions, and the gap in disease burden among different groups still exists, exacerbating the inequality of ADOD burden among regions or populations.

Therefore, in order to gain a thorough understanding of key populations affected by ADOD and to formulate targeted strategies, it is crucial and highly necessary to assess ADOD burdens across various time periods, regions, and gender demographics. This study aims to offer a comprehensive global overview of the epidemiology and risk factors of ADOD, and to understand the inequality of ADOD among regions at different development stages, so as to provide certain references for reducing the disease burden in various regions, bridging disparities between countries at different development levels, and further promoting health equity and social justice. In view of this, utilizing the Global Burden of Disease, Injuries, and Risk Factors Study (GBD) framework and estimates, we examined long-term trends in ADOD incidence, mortality, and disability-adjusted life years (DALYs) by gender in diverse regions from 1990 to 2021. Additionally, we investigated the correlation between the socio-demographic index (SDI) and the burden of the disease.

## Materials and methods

### Data source

The data used in this secondary analysis are sourced from the latest GBD study. Annual data (inclusive dates: 1990–2021) on incidence, death, DALYs, and the corresponding age-standardized rates (ASRs), as well as risk factors attributable to ADOD were searched in the Global Health Data Exchange database (http://ghdx.healthdata.org/). GBD 2021 provides location, year, age, and sex estimates of 371 diseases and injuries, 88 risk factors in 204 countries and territories from 1990 to 2021 [15]. The information from the GBD is mainly derived from household surveys, censuses, hospital records, administrative records, vital statistics, verbal autopsies, systematic literature search and so on. Details of input sources and methodology descriptions have been described elsewhere [15, 16] and data quality are globally recognized. Methods relevant to ADOD are described briefly in this section and in more detail in the Supplemental Material.

### Outcome variables

Data of estimates and their 95% uncertainty interval (UI) were collected from 1990 to 2021 by sex (both, male, and female), risk factors, and SDI region. These data were segmented by SDI quintiles, regions, countries, and territories. The SDI is the geometric mean of the lag distributed income per capita, the average educational attainment of people over 15 years of age, and the fertility rate under 25 years for a given location, with values ranging from 0 to 1, was used to position countries on the development continuum [15]. All countries and territories were sorted into five quintiles based on SDI values: high SDI region (lower bound, upper bound: 0.8103, 1.000); high-middle SDI region (0.7120, 0.8103); middle SDI region (0.6188, 0.7120); low-middle SDI region (0.4658,0.6188); low SDI region (0, 0.4658). The world is divided into 21 regions based on geographical location. To further explore the regional differences of these indicators, we used both metrics as the basis for regional grouping.

### Attributable risk factors

In this study, we focused on the burden of ADOD attributed to three specific risk factors, including smoking, high fasting plasma glucose (FPG) and high body mass index (BMI). A detailed description of the risk factors can be found in the methods section of the Supplemental Material. Both attributable number and age-standardized rate of death and DALYs of the selected risk factors were estimated using a comparative the risk assessment [17]. Assuming that exposure levels of other factors remain unchanged, the theoretical minimum risk exposure distributions of different risks were compared with the exposure distributions of a certain population, and then the population attributable fractions (PAF) of each risk were estimated. That is, the proportion of the ADOD burden due to different risk factors in the total burden in a certain population. PAF calculations utilized relative risk data, exposure data, and a theoretical minimum level of exposure.

$$PAF = \frac{{\sum }_{i}^{n}{P}_{i} \left({RR}_{i} -1\right)}{{\sum }_{i}^{n}{P}_{i} \left({RR}_{i} -1\right) +1}$$

Where, *P*_*i*_ is the percentage of the population exposed to level *i* of risks, *n* is the total number of exposure level, *RR*_*i*_ is the relative risk at level *i*. The specific methods are outlined in the previous study [17].

Based on the definition above, combined with the distribution of exposure and estimates of exposure risk for different exposure levels, ADOD mortality and DALYs attributable to risk factors were estimated. Deaths attributable to risks were calculated by multiplying the PAFs and total disease-specific deaths. DALYs were calculated as the sum of years of life lost and years lived with disability. Years of life lost were calculated by subtracting the age at death from the life expectancy for a person of that age. Years lived with disability were calculated by multiplying the prevalence of each sequela by its disability weight. For ADOD burdens attributed to risk factors, multiplying years of life lost and years lived with disability by the corresponding PAF for each factor, to obtain the attributable years of life lost and years lived with disability, and attributable DALYs can be further summed. We therefore obtained absolute number and ASRs of deaths and DALYs for ADOD due to three risk factors globally and in different SDI regions.

### Statistical analysis

To characterize the burden of ADOD in the global region, a descriptive analysis was done. The number of incident cases, deaths, and DALYs, and respective ASRs in both genders combined in different years were compared. We used the ASRs and the estimated total percentage change (ETPC) to quantify the trends of disease burden. The ASRs were age-standardized rate using the GBD world population age standard and reported per 100,000 populations, in order to compare different populations or the same population across time with different age structures.

$$ASR = \frac{{\sum }_{i=1}^{A}{a}_{i}{w}_{i}}{{\sum }_{i=1}^{A}{w}_{i}} \times 10000$$

Where *a*_*i*_ and *ω*_*i*_ represent the age-specific rate and the number of persons (or weight) of the same age subgroup in the selected reference standard population, respectively.

Joinpoint regression analysis was used to estimate the temporal trends in the ASRs of ADOD. Estimated annual percentage change (EAPC) was used to evaluate the internal trend of each independent interval of the segmentation function, and average annual percentage change (AAPC) was used to evaluate the average change trend over the whole period. Both APCs have been widely used as they are good indicators to assess the temporal trend-changes of ASRs from 1990 to 2021. The following formula was used:

$$\mathit{ln}\left(ASR\right)=\alpha +\beta x+\varepsilon$$

Where *x* is the calendar year, and APC = 100 × (*exp*(*β*)−1). Similarly, the 95% confidence interval (CI) can be obtained by the above linear regression model. APC is considered significant when it is different from 0 at the alpha of 0.05. If the value of APC and its two limits of 95%CI were both positive, then the ASR was considered to be on an upward trend within a specific period; if both values were less than 0, then the ASR was considered to be on a downward trend; otherwise, ASRs were considered to be stable.

We also used restricted cubic splines with four knots centiles to flexibly model the association of incidence, mortality, and DALY rates with SDI. Dummy variables were used for outlier regions that skewed the fit to capture the average relationship for each group, using GBD estimates from all 21 regions across all years from 1990 to 2021. Additionally, we further employed quantile regression to explore the relationship between SDI and ASRs in 204 countries and territories, in order to determine whether the impact of SDI on ADOD burden differs across quantiles. The estimate of quantile regression is based on the minimum weighted absolute value residual, with the minimum weighted absolute deviation as follows [18, 19]:

$$\text{min}\left\{{w}_{\tau } \left|{y}_{t}-a\right|\right\} = - \sum_{i:{y}_{i} <\alpha }^{T}\left(1-\tau \right) \left({y}_{t} - \alpha \right) + \sum_{i:{y}_{i}\ge a}^{T}\tau \left({y}_{t} - \alpha \right)$$

Where ${w}_{\tau }$ is the weight, *y*_*t*_ is the sum of the absolute values of the weighted deviations for any α. We selected quantile regression because it can estimate the linear relationship between different quantiles of dependent and independent variables, and can observe the changes in regression coefficients under different distributions of dependent variables in a more detailed manner, giving it an advantage over traditional linear regression models. The above statistical description and analyses were performed using the R program (Version 3.6.0).

### Ethics statement

The list of all data sources used in GBD 2021 is publicly available at the Global Health Data Exchange website (http://ghdx.healthdata.org/gbd-results-tool), therefore, ethical proof does not applicable to this study.

## Results

### Incidence of Alzheimer's disease and other dementias

In 2021, the number of global incident cases of ADOD was 9837.06 × 10^3^ (95%UI: 8620.52, 11163.70) for both sexes and all ages, and an age‐standardized rate of 119.76 (104.96,135.89) per 100,000 (Table 1). Globally, the ASIR was significantly higher in females than in males, with a male-to-female ratio of 0.78. The ASIR for women in high-middle SDI region was the highest at 145.37(127.62, 165.13) per 100,000, which was 1.27 times that of men (Figure S1). Regions with higher SDI levels generally had higher ASIR compared to areas with low SDI. The highest ASIR was 132.40(115.43,150.85) per 100,000 in the high-middle SDI region whereas the lowest rate was in low SDI area at 90.89(79.00,103.12) per 100,000. Regionally, East Asia had the highest ASIR at 149.61(129.58,171.14), while Western Sub-Saharan Africa had the lowest (73.18[63.36,83.46]), as shown in Table 1.

**Table 1:** The incidence cases and age-standardized incidence of ADOD in 1990, 2010 and 2021, and its temporal trends from 1990 to 2021

Types	1990		2010		2021		1990–2021 ETPC of ASIR (No. (95% UI))
	Incidence cases (No. × 10^3^(95% UI))	ASIR per 100,000 (No. (95% UI))	Incidence cases (No. × 10^3^(95% UI))	ASIR per 100,000 (No. (95% UI))	Incidence cases (No. × 10^3^(95% UI))	ASIR per 100,000 (No. (95% UI))	
Overall	3834.53(3367.54,4358.43)	116.97(102.77,132.32)	6824.26(5955.05,7759.76)	116.69(102.35,132.27)	9837.06(8620.52,11163.7)	119.76(104.96,135.89)	2.38(0.98,3.32)
Sex							
Male	1352.32(1177.68,1551.79)	100.69(88.05,114.43)	2491.84(2152.33,2855.69)	100.64(87.5,115.02)	3645.49(3144.74,4183.54)	103.4(89.45,118.45)	2.69(0.87,3.9)
Female	2482.21(2183.97,2820.92)	127.82(112.82,144.18)	4332.42(3807.22,4903.04)	128.39(113.25,144.89)	6191.56(5432.75,7009.23)	132.29(116.3,149.8)	3.5(2.31,4.43)
SDI region							
High SDI	1435.43(1264.75,1624.79)	127.18(112.55,142.92)	2295.03(2020.08,2588.39)	125.09(110.57,140.64)	2952.15(2586.72,3344.55)	122.61(107.45,138.44)	−3.6(−5.27,−2.08)
High-middle SDI	990.61(862.2,1138.43)	118.38(103.82,134.11)	1753.69(1520.62,2009.62)	122.54(107.17,139.4)	2582.35(2252.03,2941.77)	132.4(115.43,150.85)	11.84(10.26,13.07)
Middle SDI	844.51(735.19,963.60)	113.27(99.18,128.90)	1778.23(1544.15,2027.14)	116.45(101.79,132.53)	2902.08(2542.32,3314.77)	123.79(108.25,141.26)	9.29(7.14,10.88)
Low-middle SDI	415.38(363.22,471.14)	95.55(83.27,108.90)	750.30(655.91,851.09)	92.73(80.94,105.72)	1063.28(929.61,1207.79)	92.61(80.79,105.71)	−3.08(−3.94,−2.38)
Low SDI	144.16(125.72,163.57)	95.08(82.74,108.32)	240.20(209.70,272.10)	91.52(79.79,104.02)	328.7(287.11,372.98)	90.89(79,103.12)	−4.4(−5.35,−3.51)
Super region							
Southeast Asia, East Asia, and Oceania	944.51(814.35,1082.93)	118.86(103.61,135.31)	2078.53(1787.97,2383.61)	128.14(111.33,146.4)	3572.39(3088.51,4097.6)	141.59(122.72,161.85)	19.12(16.05,21.52)
Central Europe, Eastern Europe, and Central Asia	488.46(423.67,563.47)	115.99(101.59,132.60)	646.47(558.58,747.86)	115.98(101.28,132.53)	755.54(656.39,865.13)	113.88(99.4,129.98)	−1.82(−2.68,−0.89)
High-income	1566.39(1383.26,1776.09)	126.49(112.05,141.92)	2473.27(2177.41,2786)	123.46(109.16,138.71)	3144.43(2756.35,3576.67)	121.63(106.51,137.49)	−3.85(−5.51,−2.2)
Latin America and Caribbean	204.93(179.73,232.66)	113.41(99.39,129.12)	450.15(397.67,509.82)	112.08(98.59,127.27)	657.1(579.98,743.3)	111.22(97.62,126.41)	−1.94(−3.31,−0.7)
North Africa and Middle East	170.21(149.71,192.33)	138.06(121.37,156.98)	329.84(289.64,373.84)	134.08(117.83,152.29)	468.05(411.55,530.96)	132.19(115.75,150.35)	−4.25(−5.54,−3.17)
South Asia	315.18(273.89,359.43)	80.57(69.5,92.11)	614.36(535.36,700.62)	77.43(67.00,88.58)	924.83(799.98,1056.26)	79.00(68.26,90.52)	−1.94(−3.05,−1.05)
Sub-Saharan Africa	144.85(126.01,164.38)	97.15(84.53,110.48)	231.64(202.45,261.38)	94.65(82.56,107.45)	314.72(275.6,355.39)	93.14(81.33,105.66)	−4.13(−4.84,−3.4)
GBD Region							
East Asia	725.92(621.29,834.63)	120.29(104.75,137.02)	1670.8(1429.59,1922.54)	132.5(114.77,151.51)	2988.72(2569.17,3434.39)	149.61(129.58,171.14)	24.38(20.71,27.38)
Southeast Asia	216.53(189.08,245.89)	114.85(100.81,130.85)	403.88(352.52,460.62)	112.66(98.59,128.49)	578.24(505.93,658.51)	110.07(96.11,125.72)	−4.16(−5.28,−3.03)
Oceania	2.06(1.76,2.37)	117.17(102.35,133.78)	3.84(3.32,4.40)	113.76(98.77,130.40)	5.42(4.71,6.17)	112.05(96.98,128.81)	−4.38(−6.41,−2.43)
Central Asia	44.78(39.05,51.31)	111.72(97.73,127.69)	56.7(49.52,64.82)	111.66(97.39,127.46)	69.75(61.21,79.09)	109.82(95.87,125.3)	−1.7(−3.25,−0.32)
Central Europe	152.64(131.92,177.17)	115.01(100.69,131.62)	222.98(192.93,258.41)	113.87(99.29,130.18)	269.69(233.1,309.82)	112.42(97.97,128.46)	−2.26(−3.41,−1.08)
Eastern Europe	291.03(251.71,335.61)	117.31(102.49,133.78)	366.78(315.84,424.15)	118.10(103.10,134.88)	416.09(361.58,476.87)	115.66(100.94,131.87)	−1.4(−2.56,−0.15)
High-income Asia Pacific	212.52(185.44,242.78)	116.94(102.53,133.08)	511.36(447.89,583.44)	121.29(106.35,138.14)	701.76(614.64,802.67)	118.62(103.43,135)	1.44(−0.45,3.33)
Australasia	28.41(24.87,32.32)	123.79(108.48,139.61)	46.57(40.89,52.44)	108.97(95.79,122.17)	63.29(55.46,71.35)	105.44(92.56,118.63)	−14.82(−19.55,−10.2)
Western Europe	758.29(671.2,848.02)	122.45(108.7,136.67)	1086.07(941.12,1220.58)	118.12(103.38,132.3)	1352.81(1182.31,1543.87)	118.56(103.36,134.23)	−3.18(−5.97,−0.1)
Southern Latin America	46.98(40.64,53.79)	111.77(97.43,127.36)	79.41(69.1,91.27)	109.71(95.56,125.72)	98.31(85.03,112.62)	107.09(92.9,122.43)	−4.18(−6.61,−1.73)
High-income North America	520.2(454.54,593.02)	139.57(122.14,157.89)	749.87(657.24,850.51)	135.92(119.28,153.64)	928.26(812.57,1051.34)	131.39(114.67,149.04)	−5.86(−7.46,−4.64)
Caribbean	22.91(19.92,26.13)	97.6(85.15,110.9)	39.73(34.67,44.95)	95.67(83.43,108.46)	52.40(45.97,59.45)	95.6(83.53,108.64)	−2.05(−4.3,0.16)
Andean Latin America	14.09(12.25,15.98)	80.6(69.91,92.12)	31.34(27.23,35.96)	80.41(69.75,92.13)	44.49(38.61,50.84)	79.55(68.95,91.05)	−1.31(−4.42,1.32)
Central Latin America	74.98(65.39,85.45)	111.25(96.71,126.56)	168.82(148.27,191.57)	107.64(93.74,122.79)	248.66(218.26,282.09)	106.12(92.53,120.99)	−4.61(−5.87,−3.41)
Tropical Latin America	92.95(81.38,105.41)	129.14(113.1,146.66)	210.27(185.21,237.81)	127.98(112.63,144.95)	311.56(274.8,352.66)	126.83(111.77,144.42)	−1.79(−3.72,0.25)
North Africa and Middle East	170.21(149.71,192.33)	138.06(121.37,156.98)	329.84(289.64,373.84)	134.08(117.83,152.29)	468.05(411.55,530.96)	132.19(115.75,150.35)	−4.25(−5.54,−3.17)
South Asia	315.18(273.89,359.43)	80.57(69.5,92.11)	614.36(535.36,700.62)	77.43(67.00,88.58)	924.83(799.98,1056.26)	79.00(68.26,90.52)	−1.94(−3.05,−1.05)
Central Sub-Saharan Africa	17.48(15.15,19.94)	126.9(111.06,144.4)	32.10(28.16,36.31)	128.68(113.38,145.44)	44.06(38.63,49.58)	126.14(111.31,143.21)	−0.59(−3.02,1.79)
Eastern Sub-Saharan Africa	53.37(46.29,60.69)	107.12(93.44,121.63)	86.22(75.56,97.43)	104.26(91.13,118.09)	121.21(106.42,137.29)	102.41(89.7,116.09)	−4.4(−5.5,−3.37)
Southern Sub-Saharan Africa	24.23(21.09,27.41)	112.76(98.34,128.64)	37.18(32.25,42.31)	108.53(94.55,123.32)	47.44(41.32,54.03)	107.13(93.16,122.19)	−4.99(−6.26,−4.02)
Western Sub-Saharan Africa	49.77(43.26,56.44)	78.42(67.93,89.46)	76.14(66.34,86)	74.59(64.5,85.26)	102(88.93,115.14)	73.18(63.36,83.46)	−6.69(−7.63,−6.06)

*SDI* Socio-demographic index, *ASIR* age-standardized incidence rate, *UI* uncertain interval, *ETPC* estimated total percentage change

**Fig. 1:**
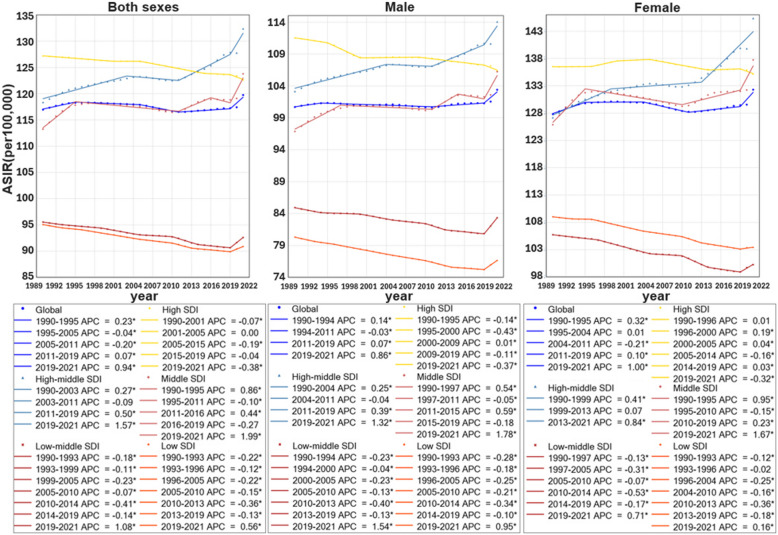
Trends in age-standardized incidence rates (per 100,000) for Alzheimer's disease and other dementias by sex, globally and in different SDI regions with Joinpoint regression

From 1990 to 2021, the global both-sex age‐standardized incidence rate increased from 116.97(102.77,132.32) to 119.76(104.96,135.89), with an AAPC of 0.06(0.05,0.07) (Table 2 and Fig. 1). Every SDI region saw a decrease in their ASIRs, with the exception of high-middle (AAPC: 0.32,95%CI: 0.27,0.38) and middle SDI (0.26[0.19,0.33]) regions. As shown in Figure S2, the ASIR was highest in high SDI regions in 1990, and the high-middle SDI region became the region with the highest ASIR by 2021 for both genders. Of the 21 regions, high-income North America ranked 1 st in 1990 and 3rd in 2021 for ASIR of ADOD, while conversely, East Asia ranked rose from 7 th to 1 st in 2021. Specific rates by regions and sexes are in Table 1.

**Table 2:** Joinpoint Analysis for ADOD-related age-standardized incidence, death rate, and DALY rate in different SDI region, 1990–2021

SDI region	Segment	ASIR		ASDR		ASR-DALYs	
		Year	EAPC(95%CI)	Year	EAPC(95%CI)	Year	EAPC(95%CI)
Global	Overall	1990–2021	0.06*(0.05,0.07)	1990–2021	0.003(−0.02,0.03)	1990–2021	0.01(−0.02,0.04)
	Trend 1	1990–1995	0.23*(0.21,0.25)	1990–1997	0.07(0.00,0.14)	1990–1996	0.12*(0.01,0.22)
	Trend 2	1995–2005	−0.04*(−0.05,−0.03)	1997–2011	−0.08*(−0.10,−0.05)	1996–2012	−0.08*(−0.11,−0.06)
	Trend 3	2005–2011	−0.20*(−0.22,−0.18)	2011–2021	0.07*(0.03,0.11)	2012–2021	0.11*(0.06,0.17)
	Trend 4	2011–2019	0.07*(0.06,0.08)				
	Trend 5	2019–2021	0.94*(0.85,1.02)				
High SDI	Overall	1990–2021	−0.12*(−0.13,−0.11)	1990–2021	−0.11*(−0.14,−0.09)	1990–2021	−0.10*(−0.12,−0.09)
	Trend 1	1990–2001	−0.08*(−0.08,−0.07)	1990–1999	−0.02(−0.05,0.02)	1990–1995	−0.002(−0.05,0.04)
	Trend 2	2001–2005	0.003(−0.04,0.05)	1999–2009	−0.22*(−0.25,−0.18)	1995–2000	−0.10*(−0.16,−0.04)
	Trend 3	2005–2015	−0.19*(−0.19,−0.18)	2009–2016	−0.001(−0.06,0.06)	2000–2010	−0.17*(−0.18,−0.15)
	Trend 4	2015–2019	−0.05(−0.09,0)	2016–2021	−0.24*(−0.32,−0.16)	2010–2017	−0.02(−0.05,0.01)
	Trend 5	2019–2021	−0.38*(−0.47,−0.28)			2017–2021	−0.20*(−0.26,−0.14)
High-middle SDI	Overall	1990–2021	0.32*(0.27,0.38)	1990–2021	0.05*(0.02,0.08)	1990–2021	0.10*(0.06,0.15)
	Trend 1	1990–2003	0.27*(0.24,0.31)	1990–2004	0.15*(0.11,0.18)	1990–2004	0.15*(0.11,0.20)
	Trend 2	2003–2011	−0.09(−0.18,0.01)	2004–2014	−0.15*(−0.22,−0.08)	2004–2013	−0.12*(−0.22,−0.01)
	Trend 3	2011–2019	0.50*(0.4,0.59)	2014–2021	0.13*(0.03,0.22)	2013–2021	0.27*(0.16,0.37)
	Trend 4	2019–2021	1.57*(0.84,2.31)				
Middle SDI	Overall	1990–2021	0.26*(0.19,0.33)	1990–2021	0.10*(0.07,0.14)	1990–2021	0.13*(0.10,0.16)
	Trend 1	1990–1995	0.86*(0.74,0.98)	1990–1996	0.14*(0.06,0.22)	1990–1995	0.36*(0.27,0.44)
	Trend 2	1995–2011	−0.10*(−0.12,−0.07)	1996–2012	−0.05*(−0.07,−0.03)	1995–2011	−0.08*(−0.09,−0.06)
	Trend 3	2011–2016	0.44*(0.27,0.61)	2012–2019	0.16*(0.08,0.23)	2011–2019	0.12*(0.07,0.17)
	Trend 4	2016–2019	−0.27(−0.82,0.28)	2019–2021	1.07*(0.62,1.53)	2019–2021	1.29*(0.92,1.66)
	Trend 5	2019–2021	1.99*(1.42,2.55)				
Low-middle SDI	Overall	1990–2021	−0.10*(−0.11,−0.1)	1990–2021	0.41*(0.31,0.52)	1990–2021	0.24*(0.19,0.30)
	Trend 1	1990–1993	−0.18*(−0.2,−0.15)	1990–1997	0.49*(0.4,0.59)	1990–1997	0.30*(0.25,0.34)
	Trend 2	1993–1999	−0.11*(−0.12,−0.1)	1997–2000	−0.004(−0.73,0.73)	1997–2000	−0.01(−0.36,0.35)
	Trend 3	1999–2005	−0.23*(−0.24,−0.22)	2000–2007	0.63*(0.51,0.76)	2000–2008	0.30*(0.25,0.35)
	Trend 4	2005–2010	−0.07*(−0.08,−0.06)	2007–2012	0.12(−0.11,0.35)	2008–2012	−0.02(−0.19,0.15)
	Trend 5	2010–2014	−0.41*(−0.43,−0.39)	2012–2015	1.13*(0.4,1.86)	2012–2015	0.55*(0.20,0.90)
	Trend 6	2014–2019	−0.14*(−0.15,−0.12)	2015–2021	0.17*(0.05,0.29)	2015–2021	0.26*(0.20,0.32)
	Trend 7	2019–2021	1.08*(1.03,1.13)				
Low SDI	Overall	1990–2021	−0.14*(−0.16,−0.13)	1990–2021	0.41*(0.32,0.50)	1990–2021	0.23*(0.16,0.29)
	Trend 1	1990–1993	−0.22*(−0.27,−0.18)	1990–1995	0.19*(0.05,0.33)	1990–1995	0.10*(0.01,0.18)
	Trend 2	1993–1996	−0.12*(−0.21,−0.04)	1995–2001	−0.18*(−0.32,−0.03)	1995–2000	−0.18*(−0.31,−0.06)
	Trend 3	1996–2005	−0.22*(−0.23,−0.21)	2001–2011	0.39*(0.33,0.45)	2000–2007	0.13*(0.06,0.19)
	Trend 4	2005–2010	−0.15*(−0.18,−0.13)	2011–2014	1.81*(1.18,2.45)	2007–2011	0.28*(0.09,0.48)
	Trend 5	2010–2013	−0.36*(−0.44,−0.27)	2014–2017	0.96*(0.34,1.59)	2011–2014	0.95*(0.55,1.36)
	Trend 6	2013–2019	−0.13*(−0.15,−0.11)	2017–2021	0.14(−0.06,0.34)	2014–2017	0.60*(0.20,1.01)
	Trend 7	2019–2021	0.56*(0.47,0.64)			2017–2021	0.19*(0.06,0.32)

*SDI* Socio-demographic index, *ASIR* age-standardized incidence rate, *ASDR* age-standardized death rate, *DALY* disability adjusted of life years, *CI* confidence interval

*P < 0.05

### Mortality and DALYs of ADOD

The number of death cases of ADOD was 1952.68 × 10^3^ (512.98, 4984.74) in 2021 globally, of which 67.9% occurred in females, and the high SDI region contributed the most (Table S1). Globally, the ASDR of ADOD decreased slightly from 25.04(6.29, 66.28) per 100,000 in 1990 to 24.9(6.36, 64.48) in 2010, then rose to 25.16(6.68, 64.25) in 2021, with an ETPC of 0.47(−3.78, 6.99) (Table S1). For SDI regions, ASDR in low-middle SDI quintile increased the fastest and experienced the highest AAPC (0.41[0.31,0.52]) over the 32-year period, as shown in Table 1 and Figure S3. From the perspective of the secular trends of sub-regions, ASDR only in high SDI region showed a downward trend in the latest segment (EAPC_2016-2021_ = −0.24[−0.32,−0.16] (Table 2). From gender perspective, the trend in the female population was more consistent with the whole population, while the trend in the male population was slightly different, especially in low and low-middle SDI regions (Table S3 and Figure S3). DALYs of ADOD also showed the similar trend with ASDR, as shown in Table S3 and Figure S4. Among all SDI quintiles, the AAPC of DALYs in the entire population and specifically in the male population, was highest in the low-middle SDI region, with AAPC of 0.24(0.19,0.30) and 0.22(0.21,0.23), respectively (Table 2 and Table S3). For the female population, the highest AAPC of DALYs was found in the low SDI region, with AAPC of 0.25(0.18,0.31) (Table S3).

### Correlations between ADOD incidence, death, DALYs and sociodemographic transition

When each regional ASIR, ASDR and DALYs for each year from 1990 to 2021 were plotted against an index of that GBD region's sociodemographic status in the same year, distinct patterns of epidemiological change were observed in Figure S5. The estimated relationship between SDI and age-standardized rates of ADOD incidence, mortality, and DALY, shown as the blue line in Figure S5, all shows obvious nonlinear relationships. As SDI increases, the incidence showed an upward trend, positive relationships were observed between ASIR and SDI in both sexes and females. ASIR in women remained stable or even slightly decreased at low SDI levels, and the increase rate accelerated after SDI reached a certain level. When SDI level was low, the ASIR for males increased rapidly, but after reaching 0.6, the growth rate slowed down, and even showed a slightly declining trend.

**Fig. 2:**
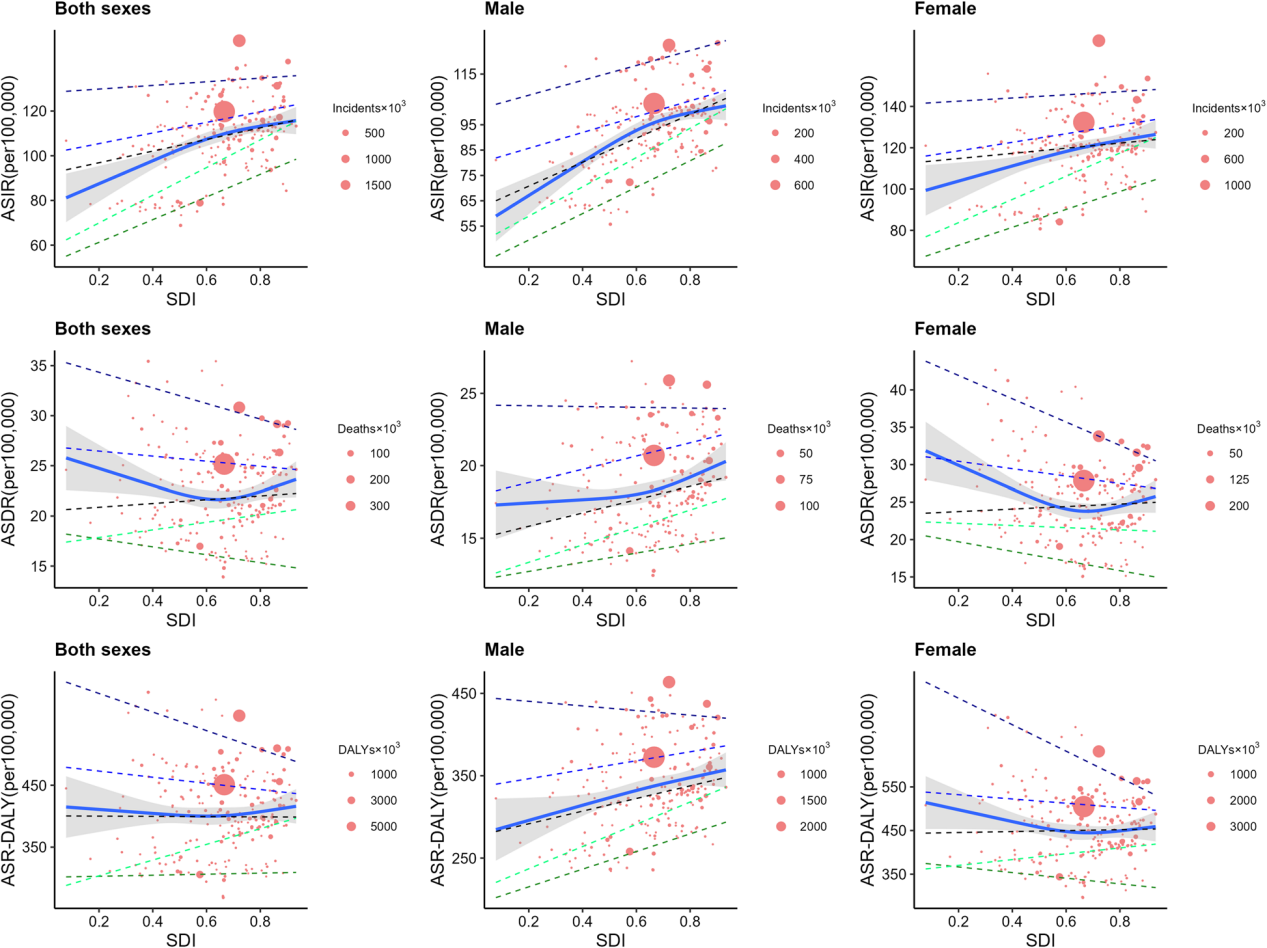
The correlation between the socio-demographic index with age-standardized incidence, death, and DALY rates of Alzheimer's disease and other dementias, in 204 countries or territories, 2021, by sex. (where circle size represents the number of incident cases, death cases, and DALYs of ADOD in 2021; the blue line and grey shadows represent the overall trends and 95%CIs in ADOD age-standardized rates associated with SDI, and the dashed lines represent quantile regression results for each quantile/95 th,75 th,50 th, 25 th, and 5 th percentiles)

We further detailed 21 regions to 204 countries to explore a more realistic association between ASR and SDI in 2021 in both genders and gender-specific populations, as shown in Fig. 2. China, with an SDI value of 0.722, has the highest number of incident cases of ADOD, followed by the United States of America (SDI = 0.862), and India (0.575), as shown in the solid circle in Fig. 2. Out of the 204 countries, 152 countries had an ASIR greater than 100 per 100,000, with 85.5% of them having SDI levels above 0.5. Among these countries, 74 countries(48.68%) belong to high or high-middle SDI quantiles.

### Risk factors attributable to ADOD burden

**Fig. 3:**
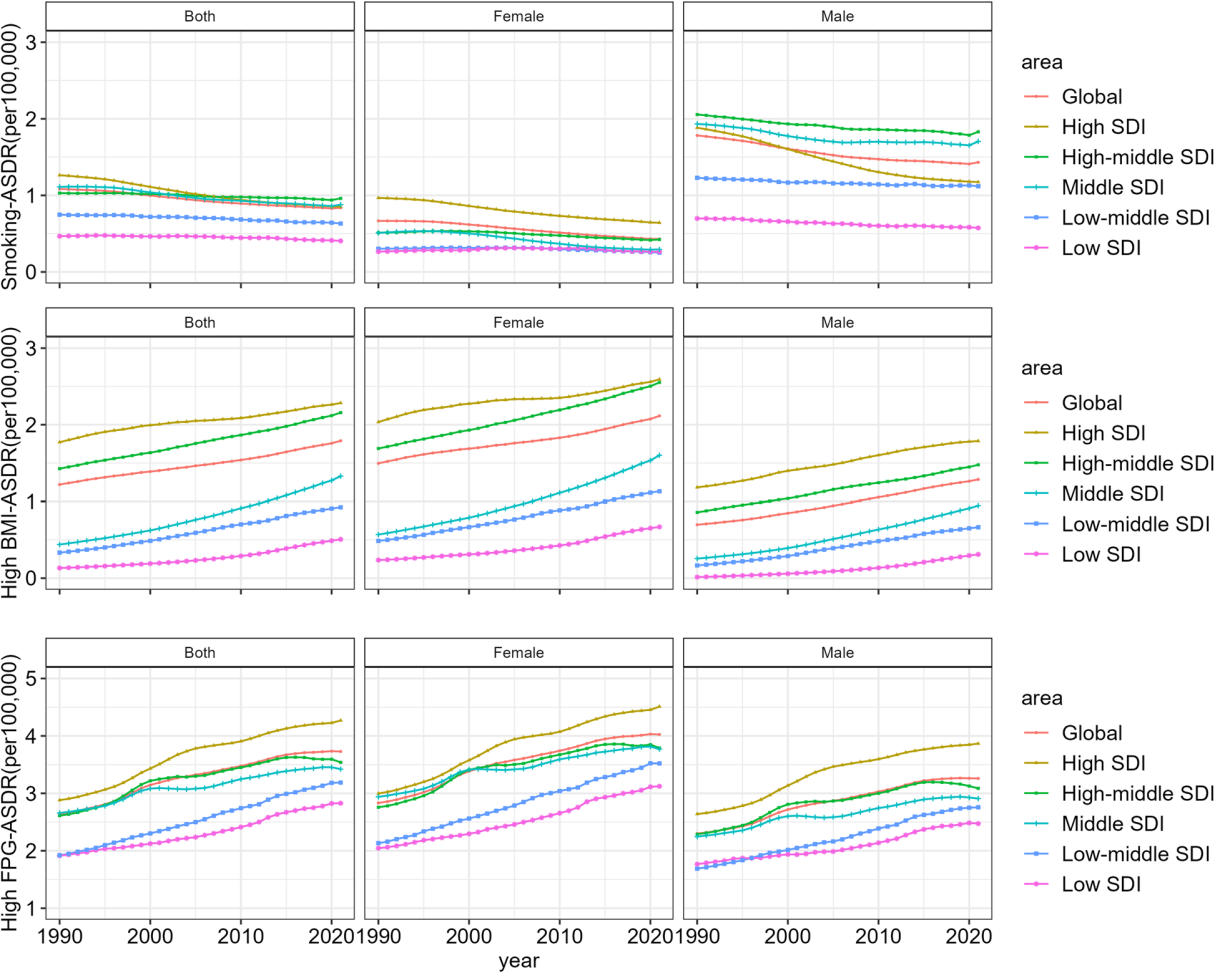
Gender-specific age-standardized death rates for Alzheimer's disease and other dementias attributable to risk factors globally and in different SDI regions, from 1990 to 2021

**Fig. 4:**
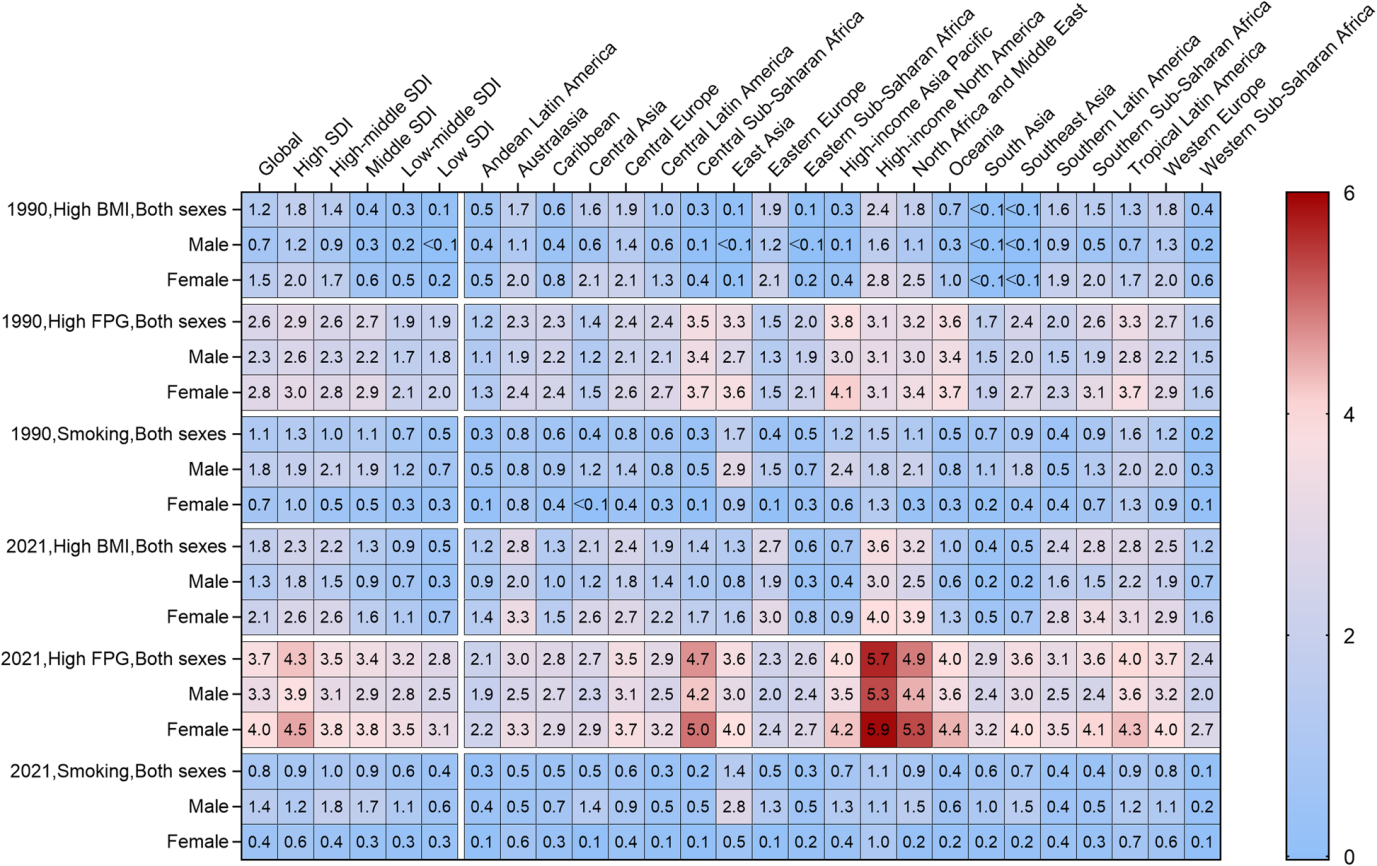
Gender-specific age-standardized death rates attributable to risk factors for Alzheimer's disease and other dementias, by global, five SDI quantiles and 21 regions, 1990 and 2021

From 1990 to 2021, both the age-standardized death and DALY rate of ADOD attributable to smoking decreased, with notable declines occurred in high SDI region (Fig. 3 and Figure S6). On the contrary, high BMI and high FPG attributable ASDRs of ADOD increased globally and in all SDI regions during the study period. The fluctuation of ASDR with high FPG attribution was the largest in high SDI region, with the heaviest burden. Figure 4 and Figure S7 specifically summarize the ASRs of deaths and DALYs by sex attributable to three risk factors in five SDI quantiles and 21 world regions in 1990 and 2021. Globally, high FPG was the leading risk factor for age-standardized deaths rates and DALYs, accounting for 3.73(0.15,11.84) and 66.42(3.83,178.85) per 100,000, respectively, in 2021, followed by high BMI, and smoking.

The attributable burden of ADOD also varied by sex both in 1990 or 2021, the ASDR caused by smoking was higher in males than in females, the ASDRs due to high FPG and BMI were much higher in females (Fig. 4). For males, ADOD burden attributed to smoking was highest in East Asia, and smallest in Andean Latin America in 2021. Across gender groups, the ADOD burden attributed to high FPG were highest in High-income North America, with ASDRs of 5.92 per100,000 in females and 5.34 per100,000 in males, respectively. Conversely, 2 regions had ASDR attributed to high BMI and high FPG less than 1.50 per 100,000. DALY rates from three risk factors among genders showed similar distribution in different regions (Figure S7).

## Discussion

ADOD is rapidly becoming one of the strongest contributors to the global burden of disease [6], placing a significant burden on both people living with ADOD, their caregivers and society. In this context, evaluating trends and changes in ADOD burden in different populations is pivotal for assessing health systems. Given the regional and gender heterogeneity in the patterns of ADOD burden, our analysis focused on the assessment the ADOD burden in different countries and genders. It offers an updated description of the trends and patterns of the incidence, mortality, and DALYs of ADOD worldwide, as well as their most relevant risk factors, and examined the association between ASRs and SDI.

Overall, the global incidence, mortality and DALYs of ADOD almost all increased slightly during the study period, especially for ASIR, suggesting that efforts to prevent and control ADOD are still needed. From a gender perspective, we found that the overall burden is higher in the female population than in males. The burden of ADOD in high SDI region was relatively heavier than that in low SDI region. Specifically, the ASIR in high SDI area was still on the rise, while the mortality and DALYs burden showed a downward trend. Although the burden was lower in regions with lower SDI levels, both age-standardized mortality and DALY rate showed an opposite upward trend compared with those in high SDI regions. Regionally, there were significant disparities among regions and countries in the three indictors, as well as the risk attributable burden. Unlike previous GBD estimates [20, 21], high FPG became the most important attributable risk factor for ADOD, especially in high SDI region. During the past 32 years, the ADOD burden caused by high BMI and high FPG showed a steady increasing trend, while the smoking attributable burden showed a downward trend, which also suggested the focus of ADOD prevention and control.

At the global and regional levels, the age-standardized incidence rates from ADOD in developed countries with high SDI levels far exceeded that in developing countries. Among the 17 regions with ASIR exceeding 100 per 100,000 populations, 14 regions had moderate or above SDI levels. Interestingly, regions with lower SDI levels tend to have poorer ADOD mortality, or outcome burden. The age-standardized rate of mortality and DALYs in high SDI area showed a sustained downward trend at different speeds. However, in regions with relatively low SDI levels, although experiencing a short-term decline, they still showed an opposite growth trend compared to regions with high SDI level as a whole, and their growth rate should not be underestimated. This suggests the growing potential of ADOD burden in low SDI region. Compared to high-income countries of the last century, low- and middle-income countries are undergoing a faster demographic transition due to an unprecedented rate of aging. Previous studies have predicted that the largest increase in ADOD is projected to occur in those countries, particularly in East Asia and Sub-Saharan Africa, where more than 70% of people with ADOD are expected to live by 2040 [22, 23].

Central Sub-Saharan Africa had the highest ADOD mortality rates among the 21 regions, with an estimated total percentage change of 14.82% from 1990 to 2021. Research indicates that limited productivity and lack of standardized treatment and diagnosis may lead to unequal access to healthcare, poor quality of medical facilities, and delayed or inadequate health services, resulting in poorer outcomes and increased disease burden for people with ADOD [24, 25]. Moreover, these regions also face basic public health challenges, including the double burden of communicable and non-communicable diseases [26]. To address the grim form of ADOD mortality in areas with lower SDI levels, prevention strategies should be strengthened for those at risk, and clinical or community management of ADOD that is feasible, effective, and affordable should be provided to individuals or families.

When using DALYs to measure the burden of ADOD, the Central Sub-Saharan Africa, including Congo and Gabon, had the heaviest burden among the 21 regions. All six countries in this region are all in the top 10 with the heaviest disease burden of 204 countries. According to a study conducted in Kinshasa-Democratic Republic of the Congo, the crude prevalence of suspected dementia was 6.2%, which was similar to other developing countries and Central African countries [27]. According to the EDAC survey in Central Africa, the prevalence of dementia was estimated at 8.1% in Bangui and 6.7% in Brazzaville, reflecting a substantial disease burden [28]. Brazil in Tropical Latin America has a high disease burden and has the 12 th highest DALY rate in the world, and previous studies have suggested that Brazil has the second highest age-standardized prevalence of ADOD globally, with an 87.7% increase in ADOD's hospitalizations over the past 10 years [29]. Among the high-income countries, Japan is a typical representative country, with heavier economic burden of ADOD. Studies have estimated that the annual economic burden in Japan is 7.404 or 12.628 trillion yen, of which 65% is public long-term care costs [30]. The prevalence of dementia in Japan is thought to have increased over time [31], possibly due to the rapid aging of the population, environmental changes, and the shift from traditional Japanese to Western lifestyles, as well as the increased prevalence of metabolic risk factors [32].

In terms of gender, the age-standardized incidence of ADOD was higher in women than in men in all SDI regions in recent decades. As the population ages, women have longer life expectancy than men, resulting in a significant proportion of the elderly population. But on top of that, we have observed that the female population also had a higher risk of age-standardized death rates compared to the male population. This suggests that differences between the sexes may not only be due to differences in longevity, but may also be influenced by other factors. Studies have reported differences in brain structure and function, genetic background, reproductive ability, hormone levels, pregnancy, menopause, and psychosocial stress responses between the sexes, which may cause differences in the epidemiology of Alzheimer's disease between the sexes [33–35]. For example, one study showed that the hippocampus shrinks faster in women, resulting in reduced hippocampal volume and abnormal nerve signal processing abilities, which may make women more vulnerable to structural and functional damage to the nervous system, increasing their risk of disease [36]. Other studies [37, 38] have linked the menopausal transition to the onset of Alzheimer's disease in mid-life based on neuroimaging outcomes, suggesting that the menopausal transition might be associated with the occurrence of Alzheimer’s disease, such that the burden of AD in post-menopausal and peri-menopausal women was higher than in pre-menopausal women and men of the same age. Specifically, compared to pre-menopause groups, peri-menopause and post-menopause groups exhibited increased Aβ deposition, reduced glucose metabolism, and reduced gray and white matter volumes in the vulnerable areas of Alzheimer’s disease. Furthermore, in most cultures, women may have lower income and education levels compared to men and are generally more likely to be primary informal caregivers in the family, and higher caregiving burden may also lead to further increases in psychological risk factors such as sleep disorders and depression in the female population, which may exacerbate the risk and consequences of the disease [35, 39]. Therefore, women with ADOD often experience a relatively heavier burden. Consequently, governments around the world should consider strengthening the prevention and treatment of ADOD as well as interventions specifically targeting women. However, we also found that the estimated annual percentage change of death and DALYs were relatively higher in males than those in females during the study period, suggesting that the disease burden in males has also rapidly increased in recent times, which means that prevention and control of male populations should not be ignored.

Based on the available risk factor data for ADOD reported in the GBD2021, we have examined three attributable risk factors associated with ADOD death, including metabolic risk factors (high BMI and high FPG) and behavioral factors (smoking). We have found that the population death patterns attributed to these risk factors have changed over the past 30 years, with regional and gender differences. GBD2019 reported that smoking was predominant among the male population, while high BMI was predominant among females [20]. Moreover, in low SDI region, the ASDR and DALYs attributable to smoking decreased slowly in women. At the same time, the burden of ADOD due to high FPG and BMI has been increasing. Notably, the burden of ASDR due to high BMI in high and high-middle SDI regions was much higher than that in middle SDI region, but the growth rate was significantly lower than that in middle SDI region. Due to the improvement of social living standards, the epidemic patterns of risk factors related to ADOD have also changed in recent years [11, 40].

The burden of ADOD caused by high BMI and high FPG has continuously increased over the past 30 years, and maintain at a high level. With the development of society, the variety of foods available to people has increased. For example, diets rich in sugar and lipids have exacerbated the development of diseases such as obesity and may lead to an increase in the incidence of metabolic diseases [41]. As the risk of obesity, social stress and physical inactivity increases in high-income countries, so does the risk of dementia[42, 43]. Generally, areas with high income usually have better medical services and more budgets to care for people with dementia. In addition, these regions often place greater emphasis on investment in educational attainment and social well-being, which can help improve mental and cognitive health, and thus reduce adverse outcomes of patients. Epidemiological studies have linked midlife obesity, as measured by anthropometric parameters such as BMI or waist-to-hip ratio, to an increased risk of dementia in later years [11, 42]. Nearly 40% of ADOD cases in the United States were associated with modifiable risk factors, with middle-aged obesity being the most important factor, followed by lack of physical activity [11]. A study has reported an association between ADOD and greater cortical atrophy in people with mild cognitive impairment [44]. Experiments have shown that a high-fat diet can promote amyloid plaque deposition and cognitive deficits in transgenic mice with Alzheimer's disease, but these effects can be effectively restored through environmental enrichment and exercise [45], which also suggests the importance of secondary prevention for high-risk populations.

With the improvement of living standards, the obesity rate in middle SDI region increased significantly. At the same time, compared with high SDI region, the construction of healthcare facilities and the health literacy level of the population in middle SDI area have not been timely and effectively improved, which may also be the reason for the growth of ASDR and DALYs in middle SDI region. High FPG was closely related to high BMI in middle SDI region, especially for women, which reflects the urgent need of local prevention and control. In low SDI region with lower development level, the burden due to high BMI may be less pronounced than in high SDI area. This may have reflected other more pressing health threats in these areas, such as infectious diseases and inadequate sanitation [46].

The burden of ADOD caused by smoking was highest in East Asia among men, and highest in High-income North America among women, the overall burden in men was more than three times higher than that in women. Tobacco causes cognitive decline and loss of brain gray matter tissue [47], and quitting smoking can reduce the progression of cognitive decline in later life. Many developed countries have implemented smoke-free laws, prohibiting the public from smoking in workplaces or specific public areas. Some countries have even enacted more comprehensive anti-tobacco laws, including restrictions on tobacco advertising and higher tobacco taxes, which have achieved considerable results [48]. The cognitive decline rate of elderly people who have successfully quit smoking has significantly slowed down in the following two years, but in low- and middle-income countries such as Guinea, the effectiveness of smoke-free legislation has been relatively less ideal [49].

Early identification of risk factors may reduce the disease burden of people with ADOD. Considering that an individual's diet and physical activity patterns are heavily influenced by environmental and social conditions, so it may be feasible to improve access to physical and social environments at a societal level for people with ADOD. In low-income countries with low levels of SDI, in addition to efforts to improve local primary health care, the establishment of community support services to support people with ADOD and their families or caregivers in the community should be strengthened. Reliable, standardized data is essential for the development of targeted interventions. As most low-and middle-income countries have incomplete information systems for neurological disorders, consideration should also be given to progressively improving their disease information systems to understand the needs of people with neurological disorders such as ADOD and their caregivers. Since people with ADOD in high-income countries have higher levels of access to community services, medicines, health products, and assistive technologies than in low-income countries [50], systematic training and education for people outside the health and social care sector can be gradually provided in these high-income countries where medical services and other security are relatively well developed [51]. From the perspective of improving national health literacy, health education on dementia prevention should be strengthened, public awareness should be raised, and healthy eating habits and exercise habits should be advocated, so as to reduce the incidence of obesity and high FPG, and further improve the local burden of ADOD in different regions.

Several limitations should be acknowledged in this study. Firstly, the reliance on data from the Global Burden of Disease estimates introduces inherent limitations associated with the GBD methodology. The methods employed to estimate missing data and adjust for measurement differences among source studies, while valuable, do not substitute for high-quality surveillance data from every country, utilizing standardized case definitions and measurement methods. The accuracy of ADOD estimates may be affected by parameters and models, particularly in certain countries. Despite efforts to address these issues, GBD's reliance on available data sources and continuous method refinement, while improving the evaluation method through iterations, may still encounter limitations in generating completely realistic results for international comparison. Secondly, the study's focus on estimated mortality and incidence rates of Alzheimer's disease and other dementias, derived through joint modeling in the GBD study, means prevalence considerations were not included in this analysis. Lastly, in the risk factor analysis, while the majority of existing risk factors in the GBD dataset were examined, not all potential risk factors for ADOD were exhaustively considered. Due to the availability of data, this study only focused on three risk factors.

In summary, the burden of ADOD is substantial and increasing, posing a challenge to the sustainability of healthcare systems. The global incidence, mortality, and DALYs of ADOD were significant disparities among regions and countries, as well as the risk-attributable burden. The incidence burden of ADOD is relatively high in high SDI region, while the death burden is relatively low but shows an increasing trend in areas with low SDI level, suggesting poor outcomes for people with ADOD in low-income countries. High FPG remains the most significant risk factor for ADOD, especially in high SDI region. Specifically, the incidence of ADOD increased more rapidly as SDI increased in areas that have historically shown lower incidence, while the incidence increased more slowly in areas with high ADOD incidence. In regions with higher mortality or DALY burden, these indicators decreased relatively faster as SDI increased. In areas where the burden of lower, the growth rate of overall burden was the largest with the increase of SDI level, suggesting that the future pressure of low-burden areas should not be ignored. The burden of ADOD is greater in the female population than in males, especially in areas with high SDI. However, in low SDI region, the burden in the male population has shown a higher trend, especially in recent years. These findings underline the importance of understanding the variations in the composition of the disease burden across countries at different stages of development, in order to implement targeted prevention and control measures in different regions or countries. Therefore, region-specific policies are needed to reduce risk factor exposure. By learning from successful disease management in certain nations, we can reduce health burdens and bridge disparities between countries with different levels of development and further promote health equity and social justice.

## Supplementary Information

Supplementary Material 1.

## Data Availability

No datasets were generated or analysed during the current study.

## Abbreviations

ADOD: Alzheimer's disease and other dementias
GBD: Global Burden of Disease Study
SDI: Socio-demographic index
DALYs: Disability-adjusted life-years
ASDR: Age-standardized death rate
UI: Uncertainty interval
BMI: Body-mass index
FPG: Fasting plasma glucose
ETPC: Estimated total percentage change
EAPC: Estimated annual percentage change
AAPC: Average annual percentage change
CI: Confidence interval
