# Supplementary material for: Epidemiological and sociodemographic transitions in the global burden and risk factors for Alzheimer's disease and other dementias: a secondary analysis of GBD 2021
Source: Int J Equity Health. 2025 May 24;24:149. doi: 10.1186/s12939-025-02530-2 (PMC12103806; doi:10.1186/s12939-025-02530-2)
Supplement: Supplementary file 1 — Supplementary Material 1. [file 12939_2025_2530_MOESM1_ESM.docx]

**Supplementary Appendix: Epidemiological and sociodemographic transitions in the global burden and risk factors for Alzheimer's disease and other dementias: a secondary analysis of GBD 2021**

**Methods**

**Disease Definition**

In this study, Alzheimer’s disease and other dementias (ADOD) were defined based on Diagnostic and Statistical Manual of Mental Disorders (DSM) III, IV or V, or revisions of International Statistical Classification of Diseases (ICD), including cognitive deficits that must include memory impairment, functional impairment, and gradual onset and continued decline. ADOD were identified according to ICD-10 with the codes F00-F03, G30 and G31, and ICD-9 codes (290, 291.2, 291.8, 294 and, 331). A progressive, degenerative, and chronic neurological disorder typified by memory impairment and other neurological dysfunctions. The definition of “death from Alzheimer’s disease and other dementias” means that ADOD are the earliest disease or injury in a series of pathological events that directly lead to death.

**Risk factors**

All of the risks in GBD were organized in five levels, where level 1 represents the overarching categories nested within level 0 risks (behavioral, environmental or occupational, and metabolic risks). Level 2 contains both single risks and risk clusters (such as the high body mass index); level 3 contains the disaggregated single risks from within level 2 risk clusters (such as smoking nested in tobacco of level 2); and level 4 has the most detailed risk categories. The GBD 2021 study incorporated the comparative risk assessment (CRA) conceptual framework previously to epidemiologically estimated and quantified the burden of 88 risk factors and associated health outcomes for a total of 631 risk-outcome pairs.

The CRA based on a causal network of hierarchically organized, potentially combined, and modifiable risks. A relative-risk model was run with MR-BRT, and a population attributable fraction (PAF) for each disease was calculated using equations. Finally, attribute burden was calculated as the PAF multiplied by total burden. In addition, to complement the RR and attributable burden estimation, the newly developed burden of proof risk function method was also applied in the new round of estimation. The mortality and DALYs of AD burden attributable to three risk factors: smoking, high fasting plasma glucose, and high body mass index were estimated by age, sex, country, and year.

**Table S1. The death cases and age-standardized death rate of ADOD in 1990, 2010 and 2021, and its temporal trends from 1990 to 2021.**

| **Types** | **1990** | | **2010** | | **2021** | | **1990-2021 ETPC of ASDR (No. (95% UI))** |
| --- | --- | --- | --- | --- | --- | --- | --- |
|  | **death cases (No.×10^3^(95% UI))** | **ASDR per 100,000 (No. (95% UI))** | **death cases (No.×10^3^(95% UI))** | **ASDR per 100,000 (No. (95% UI))** | **death cases (No.×10^3^(95% UI))** | **ASDR per 100,000 (No. (95% UI))** |  |
|  |  |  |  |  |  |  |  |
| **Overall** | 663.29(163.58, 1764.99) | 25.04(6.29, 66.28) | 1299.02(326.82, 3380.96) | 24.9(6.36, 64.48) | 1952.68(512.98, 4984.74) | 25.16(6.68, 64.25) | 0.47(-3.78, 6.99) |
| **Sex** |  |  |  |  |  |  |  |
| Male | 200.38(47.76, 550.52) | 20.18(4.98, 55.7) | 404.85(97.32, 1087.73) | 20.18(4.93, 54.33) | 626.87(153.87, 1677.85) | 20.71(5.19, 55.5) | 2.61(-2.13, 9.38) |
| Female | 462.91(115.21, 1215.53) | 27.60(6.96, 71.87) | 894.18(229.17, 2293.56) | 27.61(7.13, 70.43) | 1325.8(356.48, 3316.45) | 27.88(7.48, 69.79) | 1.00(-5.02, 9.07) |
| **SDI** |  |  |  |  |  |  |  |
| High SDI | 281.91(71.31,738.79) | 27.06(6.97,70.01) | 513.76(136.93,1287.69) | 26.43(7.02,66.23) | 719.33(196.68,1762.39) | 26.21(7.05,64.83) | -3.15(-7.6,2.06) |
| High-middle SDI | 170.71(41.72,462.49) | 25.91(6.38,69.59) | 324.83(81.74,868.54) | 26.17(6.6,69.23) | 490.09(128.59,1265.12) | 26.42(7.01,68.31) | 1.97(-4.85,11.17) |
| Middle SDI | 130.85(31.33,349.33) | 23.69(5.75,63.9) | 292.34(72.01,775.89) | 23.8(5.87,63.25) | 493.42(123.09,1282.12) | 24.56(6.3,64.53) | 3.69(-4.56,15.71) |
| Low-middle SDI | 58.42(13.86,159.85) | 17.72(4.27,49.17) | 126.63(30.24,340.92) | 19.18(4.58,52.15) | 189.59(45.9,510.23) | 20(4.85,54.05) | 12.88(5.2,23.67) |
| Low SDI | 20.73(4.97,57.26) | 19.56(4.72,54.84) | 40.28(9.45,108.62) | 20.21(4.79,55.51) | 58.64(13.91,162.64) | 22.07(5.3,61.34) | 12.84(3.88,26.25) |
| **Super region** |  |  |  |  |  |  |  |
| Southeast Asia, East Asia, and Oceania | 153.74(36.6, 409.78) | 27.62(6.67, 73.97) | 344.06(84.16, 929.28) | 27.83(6.87, 74.99) | 606.77(153.71, 1606.72) | 28.81(7.48, 76.37) | 4.32(-8.41, 21.89) |
| Central Europe, Eastern Europe, and Central Asia | 72.92(17.83, 205.45) | 21.18(5.16, 58.43) | 100.85(24.96, 282.92) | 20.92(5.13, 57.28) | 136.2(33.19, 373.24) | 20.71(5.09, 56.47) | -2.2(-6.13, 3.1) |
| High-income | 314.26(79.58, 824.73) | 27.4(7.07, 70.95) | 578.55(154.61, 1447.01) | 26.85(7.14, 67.15) | 790.48(215.97, 1928.19) | 26.43(7.1, 65.29) | -3.55(-7.85, 1.65) |
| Latin America and Caribbean | 31.16(7.65, 83.71) | 20.81(5.24, 55.49) | 80.38(20.51, 209.77) | 20.88(5.33, 54.85) | 120.71(30.81, 307.73) | 20.78(5.28, 53.39) | -0.18(-5.2, 6.33) |
| North Africa and Middle East | 26.39(6.4, 70.58) | 28.02(6.81, 75.27) | 51.94(12.9, 137.6) | 25.68(6.49, 68.18) | 73.79(18.12, 190.47) | 25.61(6.31, 66.8) | -8.61(-14.1, -2.39) |
| South Asia | 41.79(9.6, 119.8) | 14.11(3.23, 40.84) | 101.24(22.83, 277.65) | 15.85(3.71, 44.21) | 165.38(39.36, 454.12) | 17.2(4.1, 47.28) | 21.88(10.06, 39.16) |
| Sub-Saharan Africa | 23.04(5.62, 62.23) | 21.31(5.22, 58.59) | 42(10.11, 112.38) | 23.02(5.54, 62.28) | 59.35(14.29, 165.79) | 23.84(5.7, 66.18) | 11.85(2.68, 24.23) |
| **GBD Region** |  |  |  |  |  |  |  |
| East Asia | 123.42(29.18, 331.21) | 30.87(7.45, 82.33) | 279.18(68.02, 757.72) | 29.57(7.32, 79.72) | 507.66(129.18, 1368.54) | 30.41(7.81, 81.29) | -1.49(-15.14, 18.17) |
| Southeast Asia | 30.07(7.13, 80.14) | 20.67(4.97, 55.97) | 64.36(15.72, 174.2) | 22.54(5.46, 60.47) | 98.37(24.21, 259.33) | 22.64(5.56, 59.23) | 9.56(-1.43, 25.97) |
| Oceania | 0.26(0.06, 0.73) | 22.93(5.6, 63.25) | 0.53(0.13, 1.41) | 21.33(5.3, 58.09) | 0.73(0.17, 2.04) | 21.08(5.19, 59.87) | -8.09(-19.29, 5.15) |
| Central Asia | 7.26(1.79, 19.9) | 20.66(5.07, 56.11) | 8.67(2.14, 23.37) | 20.3(5.03, 54.5) | 11.02(2.73, 30.44) | 20.07(5.03, 55.69) | -2.87(-9.95, 4.08) |
| Central Europe | 22.49(5.46, 63.37) | 20.75(5.08, 56.95) | 36.24(8.98, 100.95) | 20.68(5.12, 56.21) | 49.87(12.52, 133.19) | 20.46(5.15, 54.29) | -1.41(-5.57, 4.13) |
| Eastern Europe | 43.18(10.54, 122.19) | 21.5(5.22, 59.64) | 55.94(13.84, 158.44) | 21.21(5.16, 58.53) | 75.31(18.26, 209.06) | 20.98(5.07, 57.85) | -2.39(-8.19, 5.78) |
| High-income Asia Pacific | 42.16(10.71, 109.37) | 27.79(7.27, 71.04) | 123.91(34.8, 295.41) | 26.9(7.48, 64.59) | 200.16(57.94, 463.17) | 26.59(7.46, 62.96) | -4.32(-11.46, 3.96) |
| Australasia | 5(1.23, 13.39) | 24.52(6.14, 64.68) | 10.89(2.82, 27.86) | 24.02(6.23, 61.14) | 15.25(4.01, 38.57) | 23.18(6.03, 58.68) | -5.5(-10.32, 1.26) |
| Western Europe | 153.04(38.47, 403.81) | 26.77(6.83, 69.36) | 257.74(67.19, 649.48) | 26.33(6.87, 65.94) | 339.57(90.93, 836.29) | 25.78(6.82, 64.03) | -3.7(-8.23, 1.92) |
| Southern Latin America | 7.4(1.84, 20.36) | 20.61(5.15, 55.38) | 15.03(3.82, 40.26) | 20.48(5.21, 54.79) | 18.79(4.81, 48.96) | 20.06(5.12, 52.25) | -2.65(-6.95, 3.12) |
| High-income North America | 106.65(27.64, 276.05) | 29.01(7.56, 74.58) | 170.99(45.29, 431.44) | 28.62(7.53, 72.69) | 216.71(58.26, 541.29) | 28.25(7.49, 71.28) | -2.61(-5.21, 0.5) |
| Caribbean | 3.24(0.78, 9.11) | 16.35(3.92, 44.84) | 6.73(1.68, 17.96) | 16.03(3.97, 42.98) | 9.07(2.25, 24.63) | 15.82(3.87, 43.17) | -3.28(-11.55, 6.66) |
| Andean Latin America | 2.3(0.55, 6.18) | 14.54(3.51, 39.06) | 5.29(1.27, 14.09) | 14.31(3.45, 37.92) | 7.68(1.91, 20.68) | 14.08(3.49, 37.9) | -3.19(-16.09, 14.66) |
| Central Latin America | 9.71(2.38, 26.79) | 17.16(4.23, 46.44) | 25.55(6.44, 67.98) | 16.96(4.27, 45.26) | 38.74(9.58, 102.46) | 16.84(4.16, 44.59) | -1.84(-9.31, 7.89) |
| Tropical Latin America | 15.92(3.94, 41.76) | 28.03(7.29, 73.09) | 42.8(11.13, 109.04) | 27.59(7.18, 70.73) | 65.23(17.12, 164.36) | 27.25(7.13, 68.87) | -2.77(-6.88, 2.00) |
| North Africa and Middle East | 26.39(6.4, 70.58) | 28.02(6.81, 75.27) | 51.94(12.9, 137.6) | 25.68(6.49, 68.18) | 73.79(18.12, 190.47) | 25.61(6.31, 66.8) | -8.61(-14.1, -2.39) |
| South Asia | 41.79(9.6, 119.8) | 14.11(3.23, 40.84) | 101.24(22.83, 277.65) | 15.85(3.71, 44.21) | 165.38(39.36, 454.12) | 17.2(4.1, 47.28) | 21.88(10.06, 39.16) |
| Central Sub-Saharan Africa | 2.51(0.6, 6.84) | 30.39(7.51, 79.92) | 5.48(1.32, 14.52) | 32.5(7.88, 85.73) | 8.11(1.93, 22.19) | 34.89(8.43, 93.23) | 14.82(-3.98, 38.36) |
| Eastern Sub-Saharan Africa | 8.13(1.97, 22.48) | 24.15(5.95, 66.84) | 15.53(3.72, 41.32) | 25.91(6.38, 69.75) | 23.35(5.59, 64.42) | 27.18(6.72, 73.25) | 12.54(2.23, 27.7) |
| Southern Sub-Saharan Africa | 3.88(0.95, 10.57) | 21.61(5.33, 58.95) | 6.32(1.51, 17.8) | 23.27(5.49, 64.74) | 7.86(1.89, 21.83) | 22.7(5.42, 62.82) | 5.06(-3.41, 16.82) |
| Western Sub-Saharan Africa | 8.52(2.07, 23.46) | 18.02(4.36, 49.87) | 14.67(3.48, 39.05) | 18.94(4.59, 50.95) | 20.04(4.71, 56.35) | 19.25(4.66, 53.64) | 6.86(-4.66, 21.67) |

**Note:** **ADOD: Alzheimer's disease and other dementias, SDI=Socio-demographic index, ASDR: age-standardized death rate, UI: uncertain interval, ETPC: estimated** **total percentage change**

**Table S2. The DALYs and age-standardized DALY rate of ADOD in 1990, 2010 and 2021, and its temporal trends from 1990 to 2021.**

| **Types** | **1990** | | **2010** | | **2021** | | **1990-2021 ETPC of ASR-DALYs (No. (95% UI))** |
| --- | --- | --- | --- | --- | --- | --- | --- |
|  | **DALYs (No.×10^3^(95% UI))** | **ASR-DALYs per 100,000 (No. (95% UI))** | **DALYs (No.×10^3^(95% UI))** | **ASR-DALYs per 100,000 (No. (95% UI))** | **DALYs (No.×10^3^(95% UI))** | **ASR-DALYs per 100,000 (No. (95% UI))** |  |
|  |  |  |  |  |  |  |  |
| **Overall** | 13572.31(6439.34, 29586.87) | 445.75(206.08, 958.03) | 24819.16(11737.92, 53772.59) | 443.54(207.17, 952.69) | 36332.69(17237.62, 76873.28) | 450.98(212.69, 950.16) | 1.18(-2.83, 5.15) |
| **Sex** |  |  |  |  |  |  |  |
| Male | 4465.56(2092.52, 9971.77) | 362.99(164.92, 799.27) | 8389.93(3954.27, 18774.91) | 362.94(165.48, 801.47) | 12524.13(5871.76, 27158.68) | 372.53(170.89, 805.03) | 2.63(-1.62, 7.65) |
| Female | 9106.75(4330.35, 19615.1) | 495.05(231.43, 1054.27) | 16429.23(7816.65, 35004.39) | 495.98(235.04, 1053.02) | 23808.56(11368.14, 49746.52) | 504.87(241.04, 1055.02) | 1.98(-2.88, 7.11) |
| **SDI** |  |  |  |  |  |  |  |
| High SDI | 5178.32(2446.91,11074.2) | 475.13(223.26,1007.56) | 8717.67(4167.15,18009.13) | 464.83(221.46,963.21) | 11731.99(5566.77,24033.2) | 460.75(220.37,948.43) | -3.03(-6.26,-0.4) |
| High-middle SDI | 3550.38(1664.39,7789.66) | 460.79(211.15,995.92) | 6322.35(2994.63,13982.59) | 467.45(218.63,1021.27) | 9243.77(4399.64,19638.39) | 481.7(228.79,1023.96) | 4.54(-2.19,11.55) |
| Middle SDI | 2975.64(1398.26,6593.08) | 434.43(200.09,947.97) | 6204.87(2957.39,13878.29) | 437.69(203.37,963.9) | 10141.16(4901.24,21854.76) | 455.35(215.96,982.76) | 4.82(-2.27,12.94) |
| Low-middle SDI | 1357.81(641.76,3022.1) | 334.98(157.27,731.97) | 2671.84(1226.04,5908.9) | 349.44(159.82,762.83) | 3925.27(1774.1,8561.48) | 360.4(164.09,783.93) | 7.59(1.39,14.58) |
| Low SDI | 495.71(228.39,1102.22) | 358.32(163.29,788.84) | 879.27(399.79,1944.55) | 362.43(162.66,790.54) | 1260.61(565.04,2856.16) | 383.04(167.71,863.74) | 6.9(-0.17,14.78) |
| **Super region** |  |  |  |  |  |  |  |
| Southeast Asia, East Asia, and Oceania | 3491.75(1624.9, 7799.59) | 489.15(220.21, 1071.21) | 7335.88(3493.75, 16616.86) | 500.03(232.25, 1111.84) | 12462.94(6062.46, 27133.24) | 527.36(252.46, 1151.26) | 7.81(-3.62, 20.78) |
| Central Europe, Eastern Europe, and Central Asia | 1571.93(759.97, 3438.45) | 398.4(188.43, 861.38) | 2096.93(1012.79, 4566.51) | 396.65(188.78, 851.58) | 2604.2(1227.28, 5525.01) | 391.13(184.64, 827.58) | -1.82(-4.8, 1.59) |
| High-income | 5721.32(2698.43, 12208.04) | 477.91(224.15, 1011.42) | 9637.68(4563.46, 19844.6) | 467.31(222.56, 965.49) | 12671.53(5979.74, 25859.23) | 460.55(219.82, 945.68) | -3.63(-6.84, -1.08) |
| Latin America and Caribbean | 680.86(327.88, 1482.11) | 398.02(188.33, 856.89) | 1573.48(752.71, 3338.79) | 398.15(189.3, 843.75) | 2335.28(1119.99, 4881.67) | 396.92(189.76, 829.13) | -0.28(-4.15, 3.23) |
| North Africa and Middle East | 578.98(280.43, 1264.23) | 515.8(245.62, 1112.57) | 1102.05(535.4, 2371.1) | 481.65(230.63, 1022.55) | 1566.07(753.72, 3314.93) | 476.29(225.56, 1004.2) | -7.66(-11.68, -3.43) |
| South Asia | 1009.43(467.21, 2351.86) | 272.02(126.23, 615.5) | 2190.61(980.07, 4960.93) | 289.74(129.92, 642.34) | 3450.14(1531.59, 7711.44) | 308.27(135.52, 684.96) | 13.33(4.55, 23.23) |
| Sub-Saharan Africa | 518.05(239.64, 1139.95) | 380.54(173.65, 827.08) | 882.54(402.24, 1945.55) | 397.58(176.94, 867.78) | 1242.52(557.66, 2812.33) | 406.48(177.53, 920.48) | 6.82(-0.36, 15.22) |
| **GBD Region** |  |  |  |  |  |  |  |
| East Asia | 2781.64(1277.19, 6270.51) | 527.37(233.39, 1172.37) | 5921.87(2813.45, 13375.64) | 524.91(243.92, 1168.79) | 10359.13(5080.42, 22833.68) | 555.11(267.58, 1222.86) | 5.26(-8.64, 21.47) |
| Southeast Asia | 703.42(344.01, 1485.73) | 400.2(192.5, 839.35) | 1401.61(665.43, 3023.3) | 421.11(196.54, 900.17) | 2086.18(973.51, 4362.2) | 418.63(193.3, 883.66) | 4.61(-2.99, 14.09) |
| Oceania | 6.68(3.2, 14.22) | 427.58(201.47, 930.31) | 12.39(5.99, 26.25) | 402.18(188.04, 857.98) | 17.63(8.41, 38.11) | 397.82(184.77, 863.92) | -6.96(-15.38, 2.39) |
| Central Asia | 149.21(71.67, 319.46) | 388.52(183.29, 831.85) | 182.92(89.07, 392.5) | 384.96(182.84, 814.34) | 232.27(112.59, 498.32) | 379.25(181.2, 819.07) | -2.39(-7.48, 2.61) |
| Central Europe | 488.56(236.36, 1078.06) | 391.81(185.38, 853.38) | 735.41(354.96, 1605.18) | 390.95(186.04, 843.76) | 933.2(446.02, 1970.59) | 386.04(184.19, 812.17) | -1.47(-4.63, 1.88) |
| Eastern Europe | 934.16(452.8, 2040.93) | 403.75(191.34, 870.95) | 1178.59(571.96, 2568.82) | 402.72(192.18, 864.1) | 1438.73(673.86, 3104.95) | 396.72(186.44, 853.77) | -1.74(-6.11, 3.27) |
| High-income Asia Pacific | 795.71(373.98, 1726.61) | 470.24(218.13, 1006.65) | 2037.67(977.67, 4143.22) | 466.27(224.72, 950.06) | 3028.73(1439.61, 6015.52) | 461.33(222.39, 928.28) | -1.9(-8.92, 4) |
| Australasia | 97.15(47.03, 204.5) | 443.49(212.56, 928.52) | 183.48(86, 380.47) | 419.4(197.03, 871.84) | 252.18(117.59, 522.76) | 405.09(190.47, 836.34) | -8.66(-12.98, -5.29) |
| Western Europe | 2746.24(1292.22, 5841.69) | 460.18(215.66, 971.82) | 4239.34(1989.95, 8713.14) | 449.7(213.04, 926.45) | 5385.86(2539.41, 11019) | 443.24(211.93, 909.88) | -3.68(-7.32, -0.35) |
| Southern Latin America | 150.66(73.81, 321.85) | 379.33(183.13, 803.78) | 273.05(130.68, 580.89) | 375.98(179.92, 799.88) | 340.21(162.52, 709.45) | 368.23(176.23, 766.73) | -2.93(-5.82, 0.27) |
| High-income North America | 1931.57(915.98, 4111.23) | 522.13(247.4, 1104.02) | 2904.15(1386.56, 6034.02) | 510.28(242.24, 1068.97) | 3664.55(1728.3, 7650.51) | 499.2(236.41, 1041.63) | -4.39(-6.17, -2.67) |
| Caribbean | 71.56(35.51, 153.77) | 321.56(155.29, 684.78) | 131.08(64.02, 276.5) | 315.21(154.16, 665.12) | 174.18(85.23, 368.49) | 313.56(153.61, 661.2) | -2.49(-8.21, 2.91) |
| Andean Latin America | 47.31(22.35, 102.11) | 278.53(129.89, 603.92) | 105.14(49.95, 221.64) | 275.05(129.94, 579.77) | 151.02(72.68, 318.35) | 272.02(131.15, 573.64) | -2.34(-12.09, 8.73) |
| Central Latin America | 220.4(109.63, 471.32) | 343.12(166.95, 727.8) | 522.72(255.87, 1094.69) | 338.02(164.59, 707.25) | 783.86(384.33, 1643.52) | 335.6(163.8, 703.51) | -2.19(-7.49, 3.42) |
| Tropical Latin America | 341.58(159.91, 755) | 510.43(234.39, 1109.41) | 814.54(379.63, 1741.37) | 507.12(234.54, 1081.79) | 1226.23(576.49, 2613.82) | 503.17(235.73, 1070.77) | -1.42(-4.84, 1.6) |
| North Africa and Middle East | 578.98(280.43, 1264.23) | 515.8(245.62, 1112.57) | 1102.05(535.4, 2371.1) | 481.65(230.63, 1022.55) | 1566.07(753.72, 3314.93) | 476.29(225.56, 1004.2) | -7.66(-11.68, -3.43) |
| South Asia | 1009.43(467.21, 2351.86) | 272.02(126.23, 615.5) | 2190.61(980.07, 4960.93) | 289.74(129.92, 642.34) | 3450.14(1531.59, 7711.44) | 308.27(135.52, 684.96) | 13.33(4.55, 23.23) |
| Central Sub-Saharan Africa | 62.7(28.37, 141.97) | 535.4(238.5, 1185.21) | 122.07(55.26, 267.28) | 565.35(251.34, 1227.43) | 178.4(78.7, 408.1) | 591.41(255.67, 1360.26) | 10.46(-3.41, 28.18) |
| Eastern Sub-Saharan Africa | 191.45(89.28, 426.49) | 430.46(196.38, 949.41) | 333.32(150.73, 740.91) | 448.18(199.02, 981.41) | 488.06(218.47, 1080.94) | 460.68(200.73, 1028.27) | 7.02(-0.91, 16.85) |
| Southern Sub-Saharan Africa | 81.98(39.19, 177.35) | 401.95(189.45, 867.33) | 132.68(61.53, 295.89) | 417.77(190.18, 923.97) | 168.43(77.83, 376.44) | 408.97(187.4, 904.57) | 1.75(-4.69, 10.21) |
| Western Sub-Saharan Africa | 181.91(82.65, 402.09) | 311.57(138.94, 686.95) | 294.47(133.5, 650.62) | 316.27(138.48, 692.96) | 407.63(181.17, 942.45) | 320.74(138.35, 745.09) | 2.94(-6.12, 13.02) |

**Note:** **ADOD: Alzheimer's disease and other dementias, DALY:** **Disability adjusted of life years, SDI=Socio-demographic index, ASR: age-standardized rate, UI: uncertain interval, ETPC: estimated** **total percentage change**

**Table S3. Joinpoint Analysis for ADOD-related age-standardized incidence, death rate, and DALY rate in high, high-middle, middle, low-middle, and low SDI quintiles in different sex groups for years 1990 to 2021.**

| **SDI Region** | **Segment** | **Male** | | | | | | **Female** | | | | | |
| --- | --- | --- | --- | --- | --- | --- | --- | --- | --- | --- | --- | --- | --- |
|  |  | **ASIR** | | **ASDR** | | **ASR-DALYs** | | **ASIR** | | **ASDR** | | **ASR-DALYs** | |
|  |  | **Year** | **EAPC(95%CI)** | **Year** | **EAPC(95%CI)** | **Year** | **EAPC(95%CI)** | **Year** | **EAPC(95%CI)** | **Year** | **EAPC(95%CI)** | **Year** | **EAPC(95%CI)** |
| Global | Overall | 1990-2021 | 0.074*(0.05,0.10) | 1990-2021 | 0.081*(0.05,0.11) | 1990-2021 | 0.071*(0.05,0.09) | 1990-2021 | 0.097*(0.08,0.11) | 1990-2021 | 0.021(0.00,0.04) | 1990-2021 | 0.053*(0.03,0.07) |
|  | Trend 1 | 1990-1994 | 0.145*(0.06,0.23) | 1990-2011 | -0.001(-0.01,0.01) | 1990-2009 | -0.014*(-0.02,0.00) | 1990-1995 | 0.318*(0.28,0.36) | 1990-1997 | 0.133*(0.06,0.20) | 1990-1997 | 0.163*(0.13,0.2) |
|  | Trend 2 | 1994-2011 | -0.034*(-0.04,-0.02) | 2011-2015 | 0.428*(0.29,0.56) | 2009-2019 | 0.152*(0.12,0.18) | 1995-2004 | 0.006(-0.01,0.02) | 1997-2012 | -0.062*(-0.09,-0.04) | 1997-2012 | -0.066*(-0.08,-0.05) |
|  | Trend 3 | 2011-2019 | 0.072*(0.03,0.11) | 2015-2019 | 0.021(-0.11,0.16) | 2019-2021 | 0.469*(0.14,0.8) | 2004-2011 | -0.209*(-0.24,-0.18) | 2012-2021 | 0.072*(0.03,0.12) | 2012-2019 | 0.069*(0.03,0.11) |
|  | Trend 4 | 2019-2021 | 0.858*(0.57,1.15) | 2019-2021 | 0.379*(0.11,0.65) |  |  | 2011-2019 | 0.103*(0.08,0.13) |  |  | 2019-2021 | 0.503*(0.24,0.76) |
|  | Trend 5 |  |  |  |  |  |  | 2019-2021 | 1.004*(0.83,1.18) |  |  |  |  |
| High SDI | Overall | 1990-2021 | -0.150*(-0.16,-0.14) | 1990-2021 | -0.089*(-0.11,-0.07) | 1990-2021 | -0.082*(-0.1,-0.07) | 1990-2021 | -0.030*(-0.04,-0.02) | 1990-2021 | -0.051*(-0.08,-0.02) | 1990-2021 | -0.033*(-0.05,-0.02) |
|  | Trend 1 | 1990-1995 | -0.140*(-0.16,-0.12) | 1990-1995 | -0.083*(-0.12,-0.05) | 1990-1995 | -0.076*(-0.12,-0.04) | 1990-1996 | 0.007(0,0.02) | 1990-1999 | 0.047*(0.01,0.09) | 1990-2000 | 0.047*(0.03,0.07) |
|  | Trend 2 | 1995-2000 | -0.432*(-0.46,-0.40) | 1995-2004 | -0.217*(-0.24,-0.2) | 1995-2001 | -0.294*(-0.34,-0.25) | 1996-2000 | 0.190*(0.16,0.22) | 1999-2009 | -0.133*(-0.17,-0.09) | 2000-2010 | -0.104*(-0.13,-0.08) |
|  | Trend 3 | 2000-2009 | 0.011*(0.00,0.02) | 2004-2010 | -0.161*(-0.2,-0.13) | 2001-2009 | -0.094*(-0.12,-0.07) | 2000-2005 | 0.037*(0.02,0.06) | 2009-2016 | 0.042(-0.03,0.12) | 2010-2016 | 0.032(-0.02,0.09) |
|  | Trend 4 | 2009-2019 | -0.115*(-0.12,-0.11) | 2010-2014 | 0.243*(0.16,0.32) | 2009-2018 | 0.103*(0.08,0.12) | 2005-2014 | -0.157*(-0.16,-0.15) | 2016-2021 | -0.193*(-0.29,-0.1) | 2016-2021 | -0.128*(-0.18,-0.08) |
|  | Trend 5 | 2019-2021 | -0.369*(-0.47,-0.27) | 2014-2017 | 0.062(-0.09,0.22) | 2018-2021 | -0.193*(-0.28,-0.11) | 2014-2019 | 0.030*(0.01,0.05) |  |  |  |  |
|  | Trend 6 |  |  | 2017-2021 | -0.148*(-0.20,-0.10) |  |  | 2019-2021 | -0.318*(-0.38,-0.26) |  |  |  |  |
| High-middle SDI | Overall | 1990-2021 | 0.291*(0.25,0.34) | 1990-2021 | 0.066*(0.03,0.11) | 1990-2021 | 0.121*(0.09,0.15) | 1990-2021 | 0.365*(0.3,0.43) | 1990-2021 | 0.094*(0.06,0.12) | 1990-2021 | 0.160*(0.12,0.2) |
|  | Trend 1 | 1990-2004 | 0.253*(0.23,0.28) | 1990-2003 | 0.093*(0.06,0.12) | 1990-2005 | 0.123*(0.11,0.14) | 1990-1999 | 0.410*(0.28,0.54) | 1990-2004 | 0.195*(0.16,0.23) | 1990-2004 | 0.204*(0.16,0.25) |
|  | Trend 2 | 2004-2011 | -0.04(-0.13,0.05) | 2003-2019 | -0.044*(-0.07,-0.02) | 2005-2010 | -0.130*(-0.24,-0.02) | 1999-2013 | 0.066(-0.01,0.14) | 2004-2013 | -0.112*(-0.19,-0.04) | 2004-2013 | -0.067(-0.17,0.04) |
|  | Trend 3 | 2011-2019 | 0.389*(0.31,0.46) | 2019-2021 | 0.777*(0.21,1.35) | 2010-2019 | 0.093*(0.06,0.13) | 2013-2021 | 0.840*(0.68,1) | 2013-2021 | 0.148*(0.08,0.22) | 2013-2021 | 0.337*(0.24,0.44) |
|  | Trend 4 | 2019-2021 | 1.323*(0.75,1.90) |  |  | 2019-2021 | 0.854*(0.51,1.20) |  |  |  |  |  |  |
| Middle SDI | Overall | 1990-2021 | 0.268*(0.20,0.33) | 1990-2021 | 0.166*(0.13,0.2) | 1990-2021 | 0.167*(0.14,0.20) | 1990-2021 | 0.254*(0.21,0.3) | 1990-2021 | 0.062*(0.02,0.11) | 1990-2021 | 0.113*(0.08,0.14) |
|  | Trend 1 | 1990-1997 | 0.542*(0.47,0.62) | 1990-2009 | 0.040*(0.03,0.06) | 1990-1995 | 0.257*(0.17,0.35) | 1990-1995 | 0.952*(0.82,1.09) | 1990-1996 | 0.185*(0.09,0.28) | 1990-1995 | 0.437*(0.35,0.53) |
|  | Trend 2 | 1997-2011 | -0.046*(-0.08,-0.02) | 2009-2019 | 0.211*(0.16,0.26) | 1995-2010 | 0.00(-0.02,0.02) | 1995-2010 | -0.147*(-0.18,-0.12) | 1996-2013 | -0.111*(-0.13,-0.09) | 1995-2011 | -0.125*(-0.14,-0.11) |
|  | Trend 3 | 2011-2015 | 0.589*(0.30,0.88) | 2019-2021 | 1.139*(0.61,1.67) | 2010-2019 | 0.165*(0.12,0.21) | 2010-2019 | 0.226*(0.16,0.29) | 2013-2019 | 0.141*(0.01,0.27) | 2011-2019 | 0.098*(0.05,0.15) |
|  | Trend 4 | 2015-2019 | -0.182(-0.47,0.11) |  |  | 2019-2021 | 1.209*(0.81,1.61) | 2019-2021 | 1.666*(1.04,2.3) | 2019-2021 | 0.934*(0.36,1.51) | 2019-2021 | 1.272*(0.88,1.66) |
|  | Trend 5 | 2019-2021 | 1.776*(1.18,2.37) |  |  |  |  |  |  |  |  |  |  |
| Low-middle SDI | Overall | 1990-2021 | -0.061*(-0.07,-0.05) | 1990-2021 | 0.365*(0.26,0.47) | 1990-2021 | 0.216*(0.21,0.23) | 1990-2021 | -0.169*(-0.18,-0.16) | 1990-2021 | 0.375*(0.26,0.49) | 1990-2021 | 0.211*(0.15,0.28) |
|  | Trend 1 | 1990-1994 | -0.233*(-0.25,-0.21) | 1990-2012 | 0.404*(0.38,0.43) | 1990-2021 | 0.216*(0.21,0.23) | 1990-1997 | -0.127*(-0.14,-0.12) | 1990-1996 | 0.611*(0.48,0.74) | 1990-1996 | 0.378*(0.28,0.48) |
|  | Trend 2 | 1994-2000 | -0.042*(-0.06,-0.03) | 2012-2015 | 0.84(-0.22,1.91) |  |  | 1997-2005 | -0.307*(-0.32,-0.3) | 1996-2001 | 0.001(-0.24,0.24) | 1996-2001 | -0.051(-0.23,0.13) |
|  | Trend 3 | 2000-2005 | -0.227*(-0.25,-0.21) | 2015-2021 | -0.016(-0.19,0.16) |  |  | 2005-2010 | -0.069*(-0.09,-0.05) | 2001-2008 | 0.618*(0.49,0.75) | 2001-2008 | 0.279*(0.18,0.38) |
|  | Trend 4 | 2005-2010 | -0.130*(-0.15,-0.11) |  |  |  |  | 2010-2014 | -0.525*(-0.56,-0.49) | 2008-2011 | -0.368(-1.12,0.39) | 2008-2011 | -0.196(-0.77,0.38) |
|  | Trend 5 | 2010-2013 | -0.398*(-0.47,-0.33) |  |  |  |  | 2014-2019 | -0.174*(-0.2,-0.15) | 2011-2014 | 0.933*(0.18,1.69) | 2011-2021 | 0.318*(0.27,0.36) |
|  | Trend 6 | 2013-2019 | -0.130*(-0.15,-0.12) |  |  |  |  | 2019-2021 | 0.710*(0.64,0.78) | 2014-2019 | 0.464*(0.22,0.71) |  |  |
|  | Trend 7 | 2019-2021 | 1.541*(1.47,1.61) |  |  |  |  |  |  | 2019-2021 | -0.181(-0.92,0.56) |  |  |
| Low SDI | Overall | 1990-2021 | -0.150*(-0.16,-0.14) | 1990-2021 | 0.299*(0.22,0.38) | 1990-2021 | 0.139*(0.10,0.18) | 1990-2021 | -0.169*(-0.18,-0.16) | 1990-2021 | 0.435*(0.36,0.51) | 1990-2021 | 0.245*(0.18,0.31) |
|  | Trend 1 | 1990-1993 | -0.280*(-0.33,-0.23) | 1990-2001 | -0.116*(-0.2,-0.04) | 1990-2002 | -0.141*(-0.18,-0.10) | 1990-1993 | -0.116*(-0.15,-0.09) | 1990-1992 | 0.121(-0.37,0.61) | 1990-1996 | 0.299*(0.22,0.38) |
|  | Trend 2 | 1993-1996 | -0.184*(-0.28,-0.09) | 2001-2010 | 0.275*(0.14,0.41) | 2002-2010 | 0.118*(0.03,0.20) | 1993-1996 | -0.018(-0.08,0.04) | 1992-1996 | 0.628*(0.38,0.88) | 1996-2000 | -0.304*(-0.54,-0.07) |
|  | Trend 3 | 1996-2005 | -0.246*(-0.26,-0.24) | 2010-2016 | 1.348*(1.08,1.61) | 2010-2016 | 0.713*(0.57,0.86) | 1996-2004 | -0.253*(-0.26,-0.25) | 1996-2000 | -0.452*(-0.69,-0.21) | 2000-2011 | 0.172*(0.14,0.21) |
|  | Trend 4 | 2005-2010 | -0.215*(-0.25,-0.19) | 2016-2021 | 0.006(-0.26,0.27) | 2016-2021 | 0.159*(0.02,0.30) | 2004-2010 | -0.157*(-0.17,-0.14) | 2000-2011 | 0.364*(0.33,0.4) | 2011-2014 | 1.071*(0.60,1.55) |
|  | Trend 5 | 2010-2014 | -0.339*(-0.39,-0.29) |  |  |  |  | 2010-2013 | -0.364*(-0.42,-0.31) | 2011-2014 | 1.893*(1.41,2.38) | 2014-2018 | 0.461*(0.22,0.70) |
|  | Trend 6 | 2014-2019 | -0.096*(-0.13,-0.07) |  |  |  |  | 2013-2019 | -0.180*(-0.19,-0.17) | 2014-2018 | 0.740*(0.5,0.98) | 2018-2021 | 0.028(-0.21,0.27) |
|  | Trend 7 | 2019-2021 | 0.946*(0.85,1.04) |  |  |  |  | 2019-2021 | 0.155*(0.10,0.21) | 2018-2021 | -0.017(-0.26,0.23) |  |  |

**Figure S1.** Gender-specific age standardized incidence, death, and DALY rates by global, five SDI quantiles and 21 regions, 2021.

**
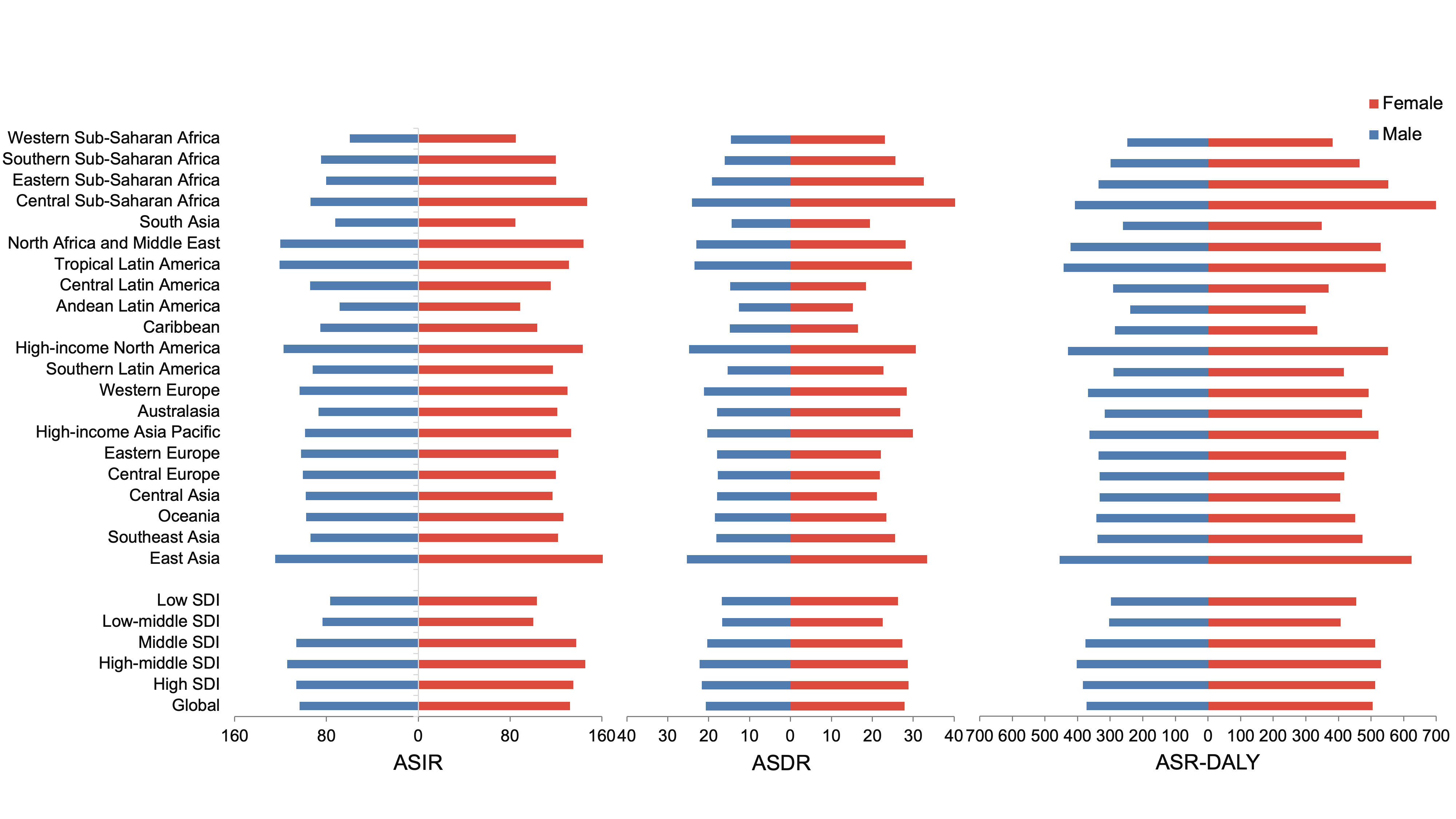
**

**Figure S2.** Age-standardized incidence, death, and DALY rates of Alzheimer's disease and other dementias, globally, and in SDI regions, from 1990 to 2021.


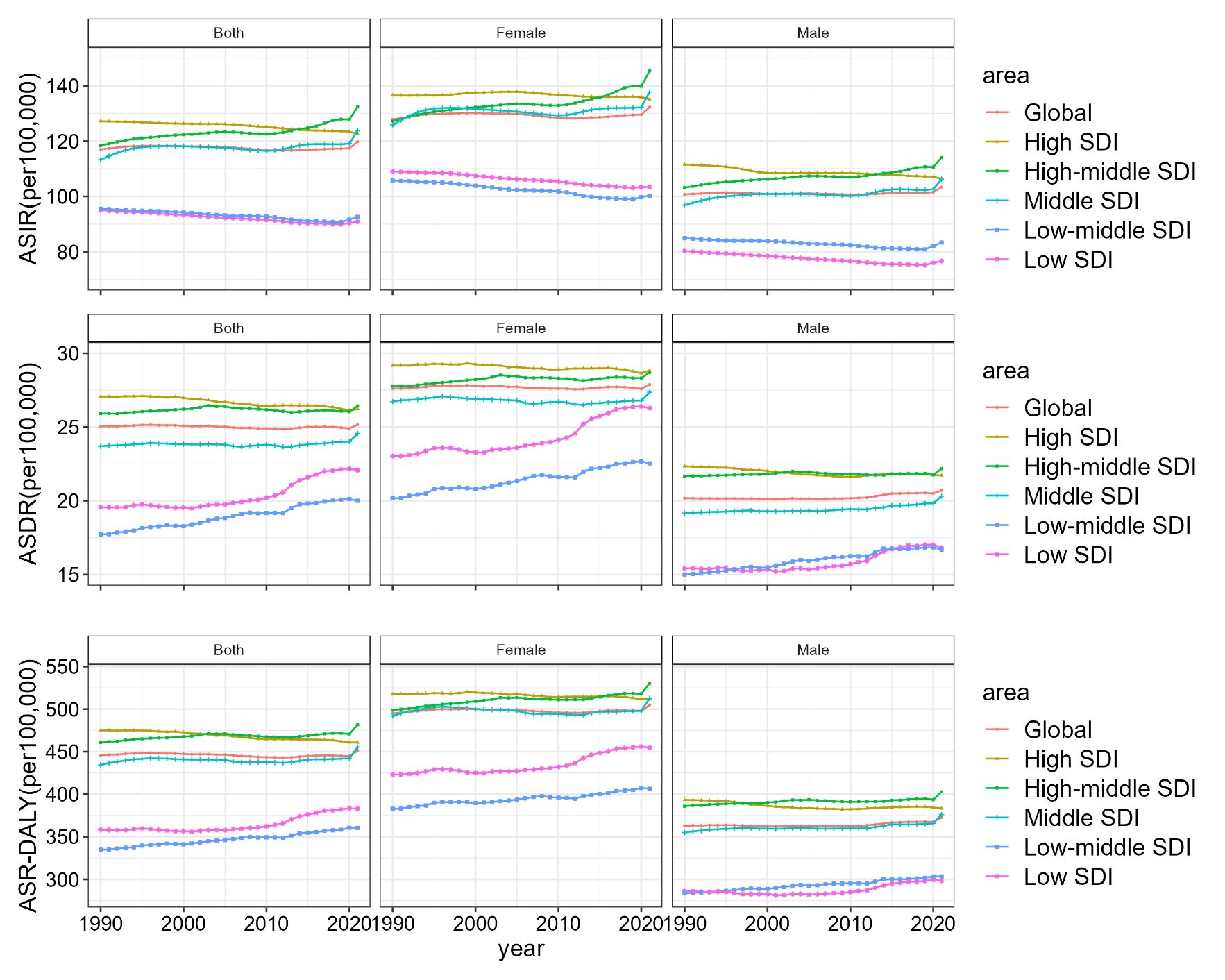


**Figure S3.** Trends in age-standardized death rates (per 100,000) for Alzheimer's disease and other dementias by sex, globally and in different SDI regions with Joinpoint regression.

**
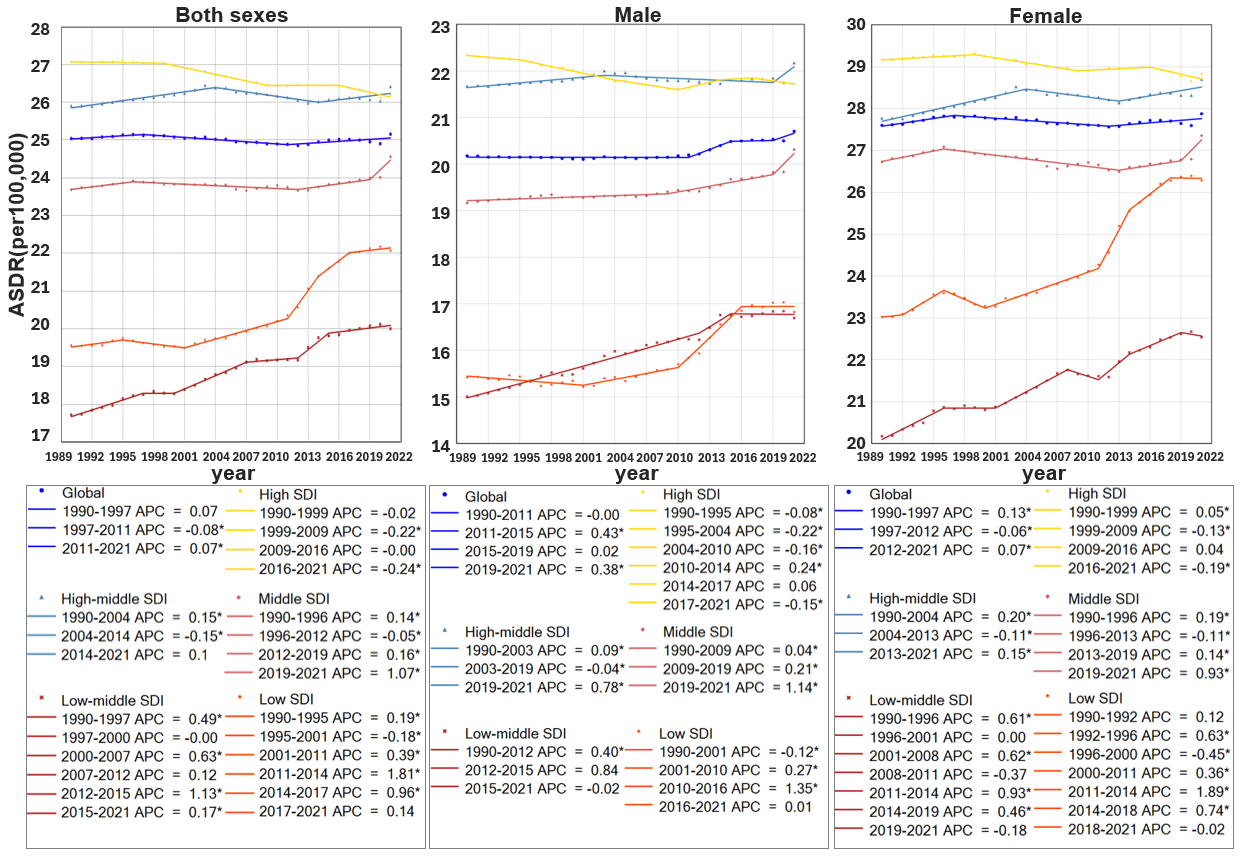
**

**Figure S4.** Trends in age-standardized DALY rates (per 100,000) for Alzheimer's disease and other dementias by sex, globally and in different SDI regions with Joinpoint regression.

**
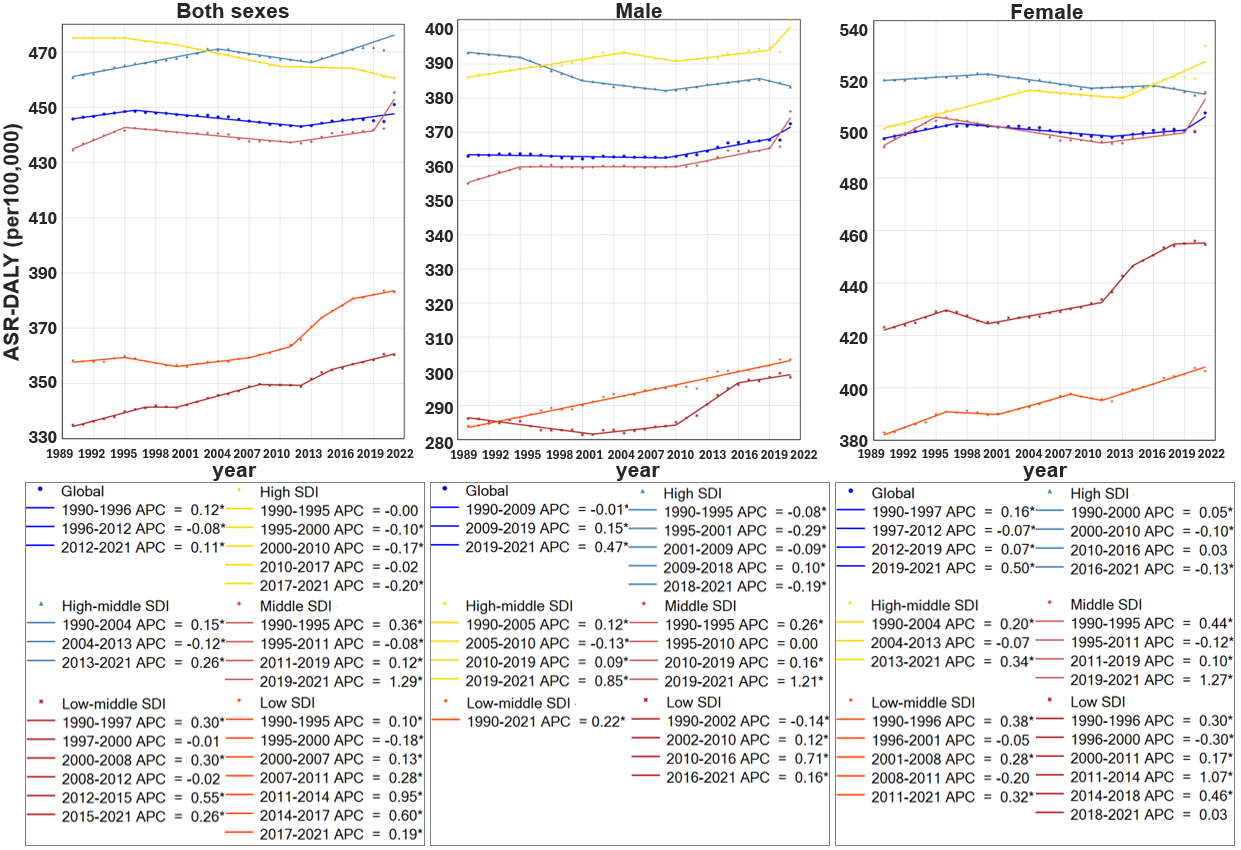
**

**Figure S5**. Relationship between the age-standardized incidence, death, and DALY rates for AD and social-demographic index (SDI) from 1990 to 2021. (Each colored line represents a time trend of the relationship for the specified region. Each point represents a specific year for that region. The blue line represents the overall global trend for the age-standardized rate of AD concerning to SDI.)

**
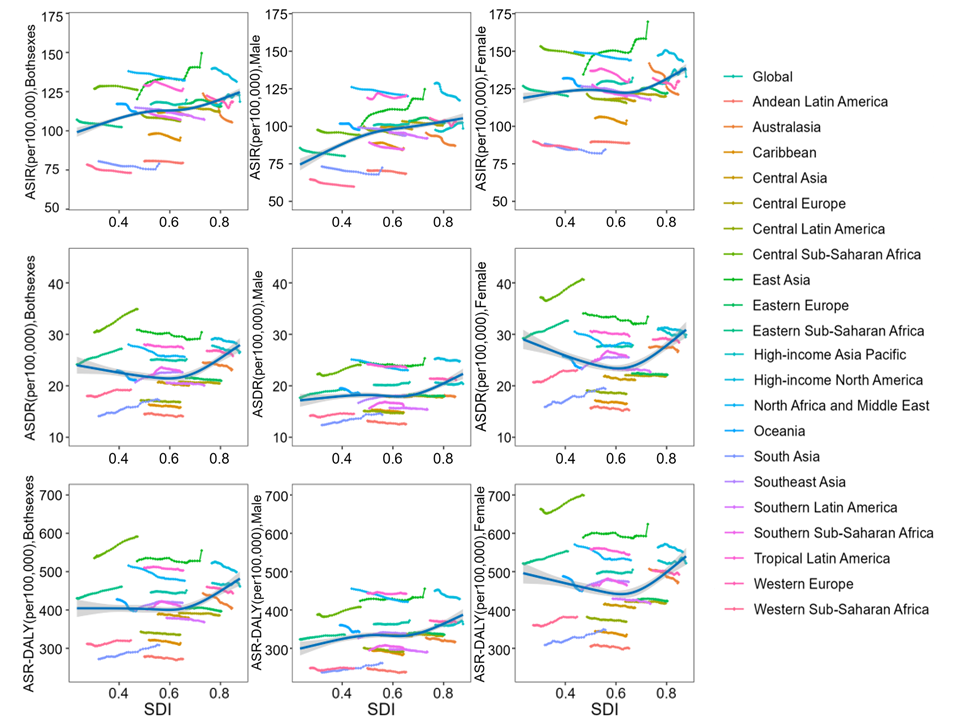
**

**Figure S6.** Gender-specific age-standardized DALY rates for Alzheimer's disease and other dementias attributable to risk factors globally and in different SDI regions, from 1990 to 2021.

**
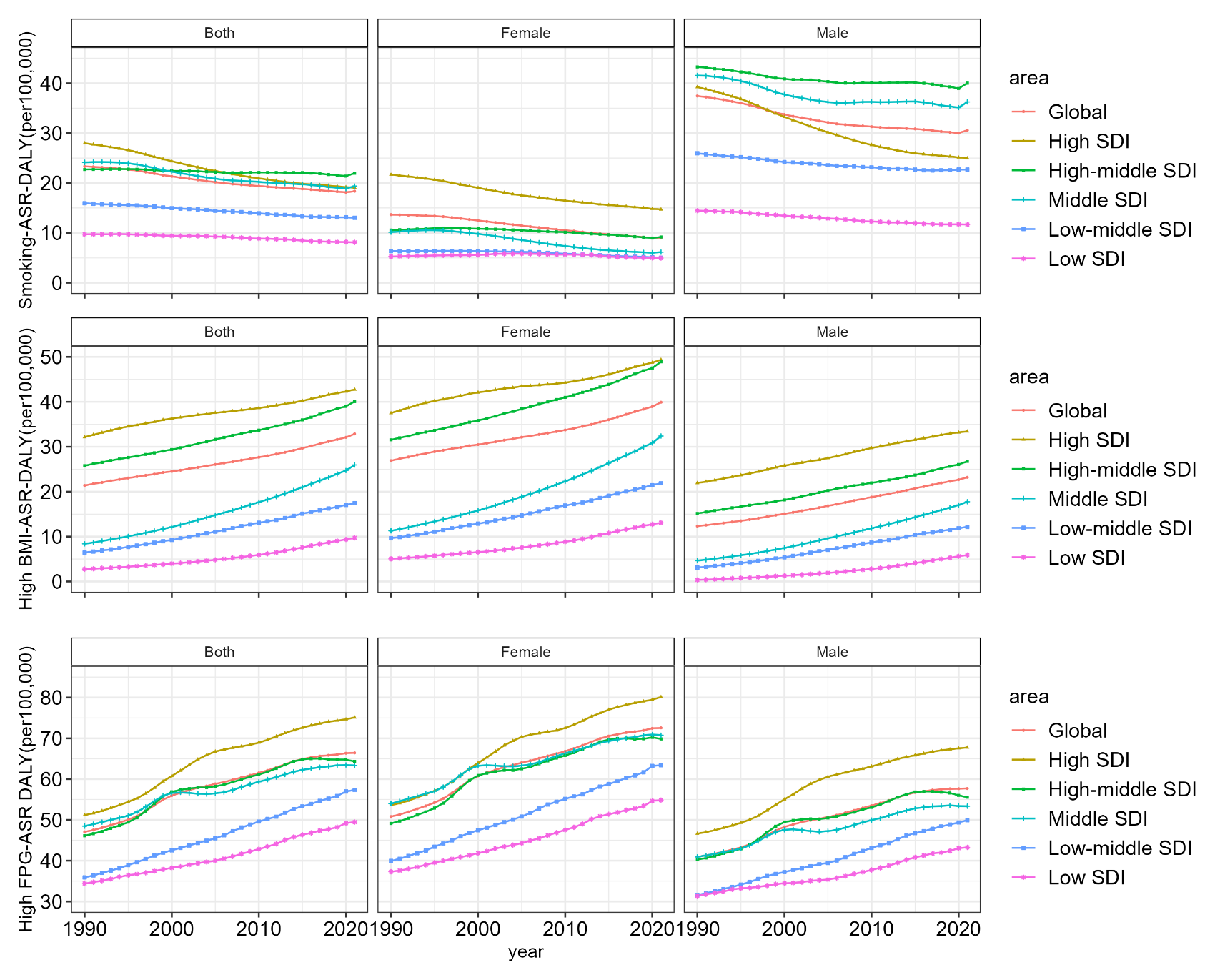
**

**Figure S7.** Gender-specific age-standardized DALY rates attributable to risk factors for Alzheimer's disease and other dementias, by global, five SDI quantiles and 21 regions, 1990 and 2021.

**
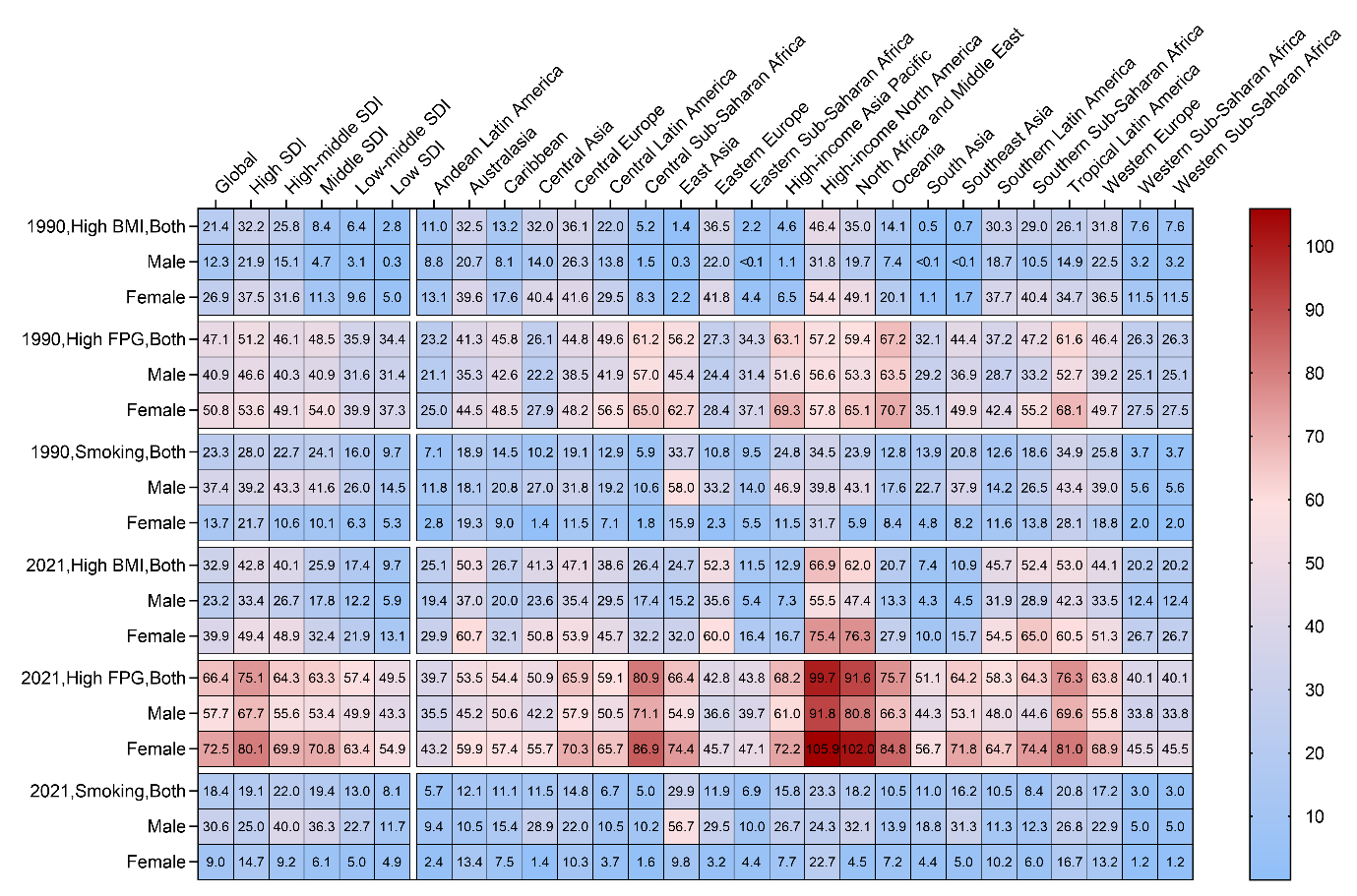
**
